# Social Protection and HIV risk Factors among Youth in Southern Africa: An Analysis of Cross-sectional Population-based HIV Impact Assessment Surveys

**DOI:** 10.1007/s10461-025-04638-6

**Published:** 2025-03-03

**Authors:** Boladé Hamed Banougnin, Delivette Castor, Joseph Baruch Baluku, Silinganisiwe Padline Dzumbunu, Oluwaseyi Dolapo Somefun, Waly Sene, David Chipanta, Lucas Hertzog

**Affiliations:** 1 United Nations Population Fund, West and Central African Regional Office, Immeuble Wolle Ndiaye, Almadies, Dakar, Senegal; 2 Division of Infectious Diseases, Department of Medicine, Vagelos College of Physicians and Surgeons, Columbia University Irving Medical Center, New York, USA; 3 Makerere University Lung Institute, Division of Pulmonology at Kiruddu National Referral Hospital, Kampala, Uganda; 4 Adolescent Accelerate Research Hub, Centre for Social Science Research, University of Cape Town, Cape Town, South Africa; 5 Centre for Addiction and Mental Health, Toronto, Canada; 6 Joint United Nations Programme on HIV/AIDS, Windhoek, Namibia; 7 Curtin School of Population Health, Curtin University, Curtin, Australia

**Keywords:** HIV risk, Social protection, Adolescents and young people, Southern african countries

## Abstract

Poverty fuels risky sexual behaviors associated with HIV infection among youth. Interventions like cash transfers may mitigate HIV risk. We explored the role of broader social protection (including food, educational, and social transfers) in reducing HIV risk among 15-24-year-olds in Southern Africa. We analyzed Population-based HIV Impact Assessment surveys data from 31,317 youth in eSwatini, Lesotho, Malawi, Namibia, Zambia, and Zimbabwe (2015–2017). Using inverse probability-weighted multivariable logistic regression, we examined associations between types of social protection and condomless sex, multiple partnerships, and high-risk sexual behaviors. Food support was associated with reduced odds of condomless sex (OR 0.71 [95% CI 0.61–0.82]), multiple partnerships (0.77 [0.63–0.95]), and high-risk sex (0.70 [0.60–0.82]). Educational support was associated with reduced odds of condomless sex (0.57 [0.46–0.59]) and high-risk sex (0.59 [0.47–0.73]). Social transfers were associated with reduced odds of condomless sex (0.62 [0.54–0.70]) and high-risk sex (0.50 [0.44–0.56]). The benefits of social protection varied across countries. Educational support was associated with reduced odds of any HIV risk factors in eSwatini, Zambia, and Zimbabwe. However, the protective effect of social transfers was only observed in eSwatini, and the benefit of food support was only significant in Namibia. Furthermore, protective associations were more pronounced among females than males. This study underscores the potential of social protection to strengthen HIV prevention efforts by mitigating poverty-related risk factors, particularly for adolescent girls and young women in Southern Africa. The impact of specific programs appears context-dependent, highlighting the need for tailored interventions.

**Supplementary Information:**

The online version contains supplementary material available at 10.1007/s10461-025-04638-6.

## Introduction

The HIV/AIDS epidemic remains a major global health issue, with sub-Saharan Africa disproportionately affected. Young people aged 15–24 years old are particularly vulnerable to HIV infection due to a complex interplay of biological, social, and economic factors [1]. In 2023, the HIV epidemic continued to disproportionately affect young women and girls. An estimated 1.9 million young women (ages 15–24) were living with HIV compared to 1.2 million young men [2]. Alarmingly, around 4,000 new HIV infections occurred among young women each week, with the vast majority of these cases (3,100) concentrated in sub-Saharan Africa [2]. In fact, 77% of all new infections in young women globally occurred in this region, emphasizing the urgent need for increased and targeted HIV prevention and support programs in sub-Saharan Africa. Youth, a developmental period, characterized by sexual exploration and increasing autonomy [3], often presents unique challenges in resource-constrained settings with limited support systems and ongoing HIV transmission [4]. Addressing this heightened vulnerability requires comprehensive and sustained interventions that empower young people to make informed choices and protect their health.

Social protection programs, encompassing diverse strategies such as cash transfers, educational support, life skills training, and food assistance, have emerged as a promising approach to mitigating the socioeconomic determinants of HIV risk [5–7]. By alleviating poverty and promoting human capital development [8], these interventions may facilitate safer sexual practices and reduce HIV incidence [5, 6, 8]. However, the evidence base on the effectiveness of social protection in influencing HIV-related outcomes remains inconclusive. While some studies indicate a protective effect [9], others highlight the limitations of conditional cash transfers and the transient nature of benefits in the absence of continued support [9, 10]. For instance, randomized controlled trials in Malawi demonstrated a reduction in HIV prevalence among adolescent girls receiving cash transfers, but these gains dissipated after the intervention concluded [10]. Observational studies in South Africa and Tanzania have further explored the potential of social protection to reduce risky sexual behaviors [11, 12].

Young people face significant barriers in accessing essential HIV prevention services, including pre-exposure prophylaxis (PrEP), a critical component of comprehensive HIV prevention strategies. While PrEP availability has been increasing in Southern Africa, access for young people remains uneven across the region. In Eswatini, despite increased PrEP availability at youth-friendly clinics, particularly from 2021 onwards, initial uptake was slow due to stigma and limited awareness [13]. Lesotho has made significant strides since 2016 in expanding PrEP access through community-based programs, with PrEP provided free of charge at public healthcare facilities. However, challenges such as stigma, limited awareness, and access barriers in remote areas may still hinder uptake among young people [14]. Malawi, despite integrating PrEP into its national HIV program in 2016, continues to face service delivery challenges, particularly in rural areas, which limit access for adolescents [15]. Similarly, Namibia, although prioritizing PrEP for key populations, including young people, in its 2017 national HIV strategy, still grapples with uneven geographical access and service availability [16, 17]. Zambia, while scaling up PrEP services since launching its national PrEP program in 2017, faces social and cultural barriers, including parental consent requirements, that restrict access for adolescents [18]. Zimbabwe, despite including PrEP in its national HIV strategy in 2017, has experienced slow implementation, resulting in limited access for young people [13]. Understanding these country-specific PrEP landscapes is crucial for informing broader HIV prevention efforts, including those involving social protection.

Social protection programs in Southern Africa have evolved over time, shaped by diverse historical and socioeconomic contexts. While varying in composition and coverage, these programs generally aim to mitigate poverty and vulnerability, particularly among children and families. In eSwatini, social protection efforts have historically focused on cash transfers, primarily through the Orphaned and Vulnerable Children (OVC) grant, which has seen significant expansion in recent years [19]. Lesotho boasts a long-standing Child Grants Programme, providing cash assistance to vulnerable households with children, and has recently expanded its social protection portfolio to include public works programs and old-age pensions [20]. Malawi has pioneered social cash transfer programs, notably the Malawi Social Cash Transfer Programme, which targets ultra-poor households and has demonstrated positive impacts on poverty reduction and child well-being [21]. Namibia has implemented a comprehensive social safety net, including a universal old-age pension, disability grants, and targeted food assistance programs [22]. Zambia has made strides in expanding social protection coverage, with its Social Cash Transfer Scheme reaching a growing number of vulnerable households [23]. Zimbabwe has implemented various social protection initiatives, including the Harmonized Social Cash Transfer program, which provides cash assistance to vulnerable households, and the Basic Education Assistance Module, which supports access to education for orphans and vulnerable children [24].

Despite these efforts, social protection coverage remains uneven across the region, and significant challenges persist in reaching the most vulnerable populations, particularly adolescents and young people. Furthermore, a comprehensive understanding of the complex relationship between social protection and HIV risk among youth remains lacking. Existing research is often limited to smaller-scale cash transfer interventions, lacking generalizability and a nuanced understanding of the impact of diverse social protection program types. This gap in knowledge underscores the significance of our multi-country study, which utilizes large-scale nationally representative data to provide robust evidence on the association between three types of social protection (social transfers, educational support, and food support) and HIV risk behaviors among adolescents and young people. To our knowledge, this is the first study to offer such a comprehensive, cross-national examination of the relationship between various social protection measures and HIV risk factors in this population.

This study seeks to provide contemporary and compelling evidence of the crucial role that social (cash) transfers, educational support, and food assistance may play in mitigating HIV risk among youth in six Southern African countries (including eSwatini, Lesotho, Malawi, Namibia, Zambia, and Zimbabwe). It examines the associations between these types of social protection and HIV risk factors among adolescents and young people, exploring gender differences in these relationships. By offering a multi-country perspective, this research aims to inform regional policies and programs designed to combat the HIV epidemic, leveraging social protection as a tool for HIV prevention.

## Methods

### Study Population, Setting and Data Collection

We obtained publicly available cross-sectional Population-based HIV Impact Assessment (PHIA) surveys data from six Southern African countries: eSwatini (2016–17), Lesotho (2016–17), Malawi (2015–16), Namibia (2017), Zambia (2016), and Zimbabwe (2015–16). PHIA surveys measure HIV prevalence, recency, risk factors and antiretroviral treatment outcomes in priority countries with high HIV burden supported by The President’s Emergency Plan for AIDS Relief (PEPFAR) [25]. PHIAs also collected sociodemographic data on receipt of various forms of economic support in all six surveys, allowing for standardised measures across countries. The PHIAs used two-stage sampling based on each country’s national census enumeration areas and household information to select representative samples of adults and children. Households were randomly selected from primary sampling units comprised of census-derived enumeration areas. Within selected households, all eligible adults aged 15 years or older were included. Within a random subgroup of half of sampled households, adults provided data on all children aged 0–14 years. This study focuses on adolescents and young people aged 15–24 years. Detailed information about survey design, sampling methods, and refusal rates is available in the PHIA final survey reports [26].

### Outcome Variables

We assessed three measures of HIV risk factors in the twelve months prior to the survey: condomless sex, multiple sexual partnerships, and high-risk sex. *Condomless sex* was defined as self-reported non-use of condom during sexual intercourse with at least one of the three most recent sexual partners (if any) in the past twelve months. *Multiple sexual partnerships* was defined as self-reported sexual intercourse with at least two partners in the past twelve months. *High-risk sex* was a composite measure defined as condomless sex with either multiple partners, sexual intercourse in exchange of money or material support, or sexual intercourse with partner(s) five or more years older than the participant. All three measures of HIV risk factors are binary.

### Variables of Interest

In participating households, respondents were asked whether their households received any forms of external economic support in the last three or twelve months. In all six countries, external economic support questions included cash transfer (e.g., pensions, disability grants, child grant), assistance for school fees, material support for education (e.g., uniforms, schoolbooks, education, tuition support, bursaries), income generation support in cash or kind (e.g., agricultural inputs), food assistance provided at the household or external institution, and social pension. Data on material or financial support for shelter was not collected in eSwatini and data on remittances was collected only in Lesotho. These (two) measures were not included in the present analysis to facilitate comparison across countries. Based on evidence from the 2021–2025 Africa Regional Social Protection Strategy [27], we assessed three measures of social protection: social transfer, educational support, and food support. *Receipt of social transfer* was defined as self-reported receipt of cash transfer, income generation support in cash or kind, or social pension. *Receipt of educational support* was defined as self-reported receipt of assistance for school fees or material support for education. *Receipt of food support* was defined as self-reported receipt of food assistance provided at the household or external institution. All three measures are binary.

### Main Confounding Factors

The literature suggests that households who receive social protection may differ systematically from those who do not receive social protection in terms of socioeconomic characteristics [27, 28]. The majority of social protection programmes in Sub-Saharan African countries target poor households and school-aged children in resource-poor settings [29–32]. In line with that empirical evidence, we included two confounding factors: household wealth index and number of children aged 0–17 years in the household. Household wealth index was determined by applying principal component analysis (PCA) to a common set of household characteristics and assets ownership (separately for urban and rural households). Scores obtained from PCA were categorised to obtain wealth quintiles.

### Statistical Methods

All analyses were done in Stata 18 using survey weights including base weights and jackknife (JK) replicate weights [33]. Weights were based on sampling probabilities and adjusted for non-response and post-stratification to national population projections from the survey year based on age and sex [25]. In addition, we adjusted variance estimates with JK variance estimation [34]. In pooled data analyses, we combined the replicate weights variable across countries. We calculated descriptive frequencies and 95% confidence intervals (CI) for sociodemographic characteristics, receipt of social protection, and HIV risk factors. We computed differences in the prevalence of individual HIV risk factors between youth in household with and without educational support, food support, and social transfer using chi-square tests of independence.

We estimated the associations between receipt of (each type of) social protection and individual HIV risk factors using covariate-adjusted logistic regression weighted by the inverse probability of social protection receipt. Inverse probability weights were estimated using probit regression adjusting for the main confounding factors for each type of social protection and by country. This technique is similar to that of propensity score estimation widely used to evaluate the effects of social programmes on health outcomes in non-randomised studies [12, 35, 36, 37], but adequately accounts for the PHIA complex survey design elements (including weighting, clustering, and stratification) to produce weighted association measures and adjusted variances.

We tested whether the associations of individual HIV risk factors with each type of social protection differ for females and males (i.e., sex interaction effect) in three steps. First, we fit logistic regression models adjusting for each type of social protection and their interaction with sex, and covariates. Second, using post-regression modelling, covariate-adjusted average marginal effects (AME) were estimated to plot the differences in the probability of reporting HIV risk factors between youth in households with and without social protection for females and males. Third, sex interactions were tested using second difference (i.e., difference in AME across males and females) tests [38, 39].

We reported estimates for each country and pooled data. Demographics such as place of residence (rural vs. urban), age (15–17 vs. 18–24 years), educational level (primary, secondary or higher vs. no education), marital status (ever married or living with a partner vs. never married or lived with a partner) were included as covariates in country-specific regression models. Country fixed effect was added as covariate for the pooled data regression models. We did not impute missing data because the number of participants with missing data was small, and we assumed that missing data for variables would occur at random.

### Ethical Considerations

The data is deidentified and can be accessed at the PHIA website with a brief request (at https://phia-data.icap.columbia.edu/datasets). Local ethics committees, Ministries of Health, Institutional Review Boards at the Center for Disease Control and Prevention (Atlanta), and Columbia University Medical Center (New York) approved the PHIA surveys in each country. All participants (and minors’ caregivers) provided written informed consent. Anonymized data were used for statistical analyses.

### Role of the Funding Source

The funder of the study had no role in study design, data collection, data analysis, data interpretation, or writing of the report.

## Results

This analysis included 37,317 young women and men aged 15–24 years (3,797 in eSwatini, 4,420 in Lesotho, 7,171 in Malawi, 6,098 in Namibia, 8,089 in Zambia, and 7,742 in Zimbabwe). The pooled analytic sample is described in Table 1, with country-specific descriptions provided in the appendix (p. 4). Slightly over half (50.7%) of the sample were female, and the majority (67.7%) were between 18 and 24 years old. Most resided in rural areas (65.0%), were currently in a relationship (96.5%), and had attained at least a primary level of education (41.6% primary, 56.0% secondary or higher).

Social protection coverage was generally low, with only 16.2% of youth reporting any form of social protection (8.8% social transfers, 4.5% educational support, 6.0% food support). Namibia and eSwatini had notably higher coverage compared to the other four countries (see appendix p. 4 for details). Social transfer coverage ranged from 3.7% in Zambia to 31.9% in Namibia. eSwatini had the highest proportion of youth receiving educational support (25.3%), while Zambia had the lowest (2.5%). Similarly, food support was most prevalent in eSwatini (22.2%) and least prevalent in Zambia (1.7%).

Among the youth surveyed, 34.4% reported condomless sex in the past 12 months (Table 1). This prevalence was highest in Malawi (39.9%) and lowest in Eswatini (17.0%). Multiple sexual partnerships were reported by 9.0% of the pooled sample, ranging from 8.3% in Zimbabwe to 18.7% in Lesotho. Finally, 17.8% of youth engaged in high-risk sexual behaviors in the past 12 months, with prevalence ranging from 10.8% in Eswatini to 19.4% in Malawi.

**Table 1:** Characteristics of youth aged 15–24 years in six southern African countries (pooled data)

	*n*	Weighted % (95% CI)
Sex	37,317	
Male		49.3 (49.2–49.4)
Female		50.7 (50.6–50.8)
Place of residence	37,317	
Rural		65.0 (63.4–66.7)
Urban		35.0 (33.3–36.6)
Participant’s age	37,317	
15–17		32.3 (31.8–32.8)
18–24		67.7 (67.2–68.3)
In a relationship	37,317	96.5 (96.2–96.7)
Education attainment	37,305	
None		2.4 (2.2–2.6)
Primary		41.6 (40.6–42.6)
Secondary or higher		56.0 (54.9–57.0)
Receipt of any social protection	37,317	16.2 (15.5–17.1)
Social transfer	37,317	8.8 (8.3–9.4)
Education support	37,317	4.4 (4.0–4.9)
Food support	37,317	6.0 (5.5–6.6)
Condomless sex	36,235	34.4 (33.7–35.2)
Multiple sexual partnerships	36,390	9.0 (8.6–9.4)
High risk sex	36,235	17.8 (17.3–18.3)

**Fig. 1:**
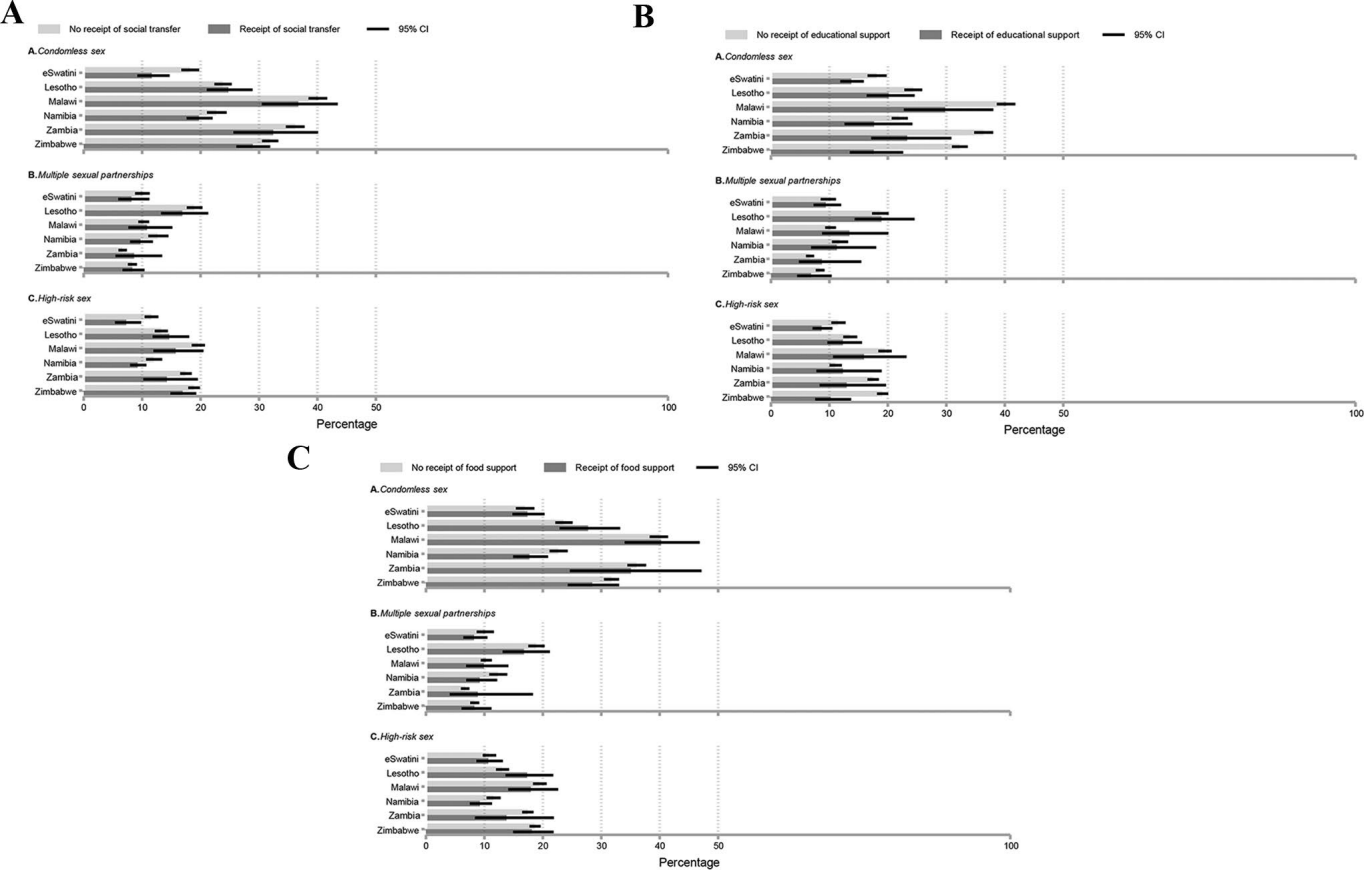
Country-specific weighted prevalences of HIV risk factors (for **A**. condomless sex, **B**. multiple sexual partnerships, and **C**. high-risk sex) between youth in households with and without each type of social protection (including social transfer, educational support, and food support) Note. p values obtained from Chi-2 tests of associations between receipt of social protection and HIV risk factors are reported in the appendix (p. 5)

Table 2 displays the pooled bivariate descriptive associations of HIV risk factors with social transfer, educational support, and food support. Figure 1 shows corresponding findings by country, and the appendix (p. 5) reports chi-square test statistics data. In the past 12 months, condomless sex and high-risk sex were significantly less frequent among youth in households with social transfers, compared to youth in households without social transfers (Table 2). Youth in households with educational support reported significantly less condomless sex and high-risk sex in the past 12 months, compared with youth in households without educational support. Additionally, youth in households with food support were significantly less likely to report condomless sex in the past 12 months than those in households without food support.

**Table 2:** Weighted prevalences (and 95% CIs) of HIV risk factors (condomless sex, multiple sexual partnerships, and high-risk sex) between youth in households with and without each type of social protection (social transfer, educational support, and food support) in Southern Africa (pooled data)

	Social transfer			Educational support				Food support	
	No receipt	Receipt	p value [chi-2]	No receipt	Receipt	p value [chi-2]	No receipt	Receipt	p value [chi-2]
Condomless sex	35.0 (34.2–35.8)	28.4 (26.4–30.5)	< 0.001 [169.7702]	35.1 (34.3–35.8)	21.0 (18.3–24.0)	< 0.001 [406.3188]	34.7 (33.9–35.5)	30.2 (27.5–33.0)	0.005 [55.3476]
Multiple sexual partnerships	9.0 (8.6–9.4)	9.5 (8.3–10.8)	0.362 [3.1985]	8.9 (8.5–9.4)	10.2 (8.4–12.3)	0.182 [9.1776]	9.0 (8.6–9.4)	9.4 (8.0–11.0)	0.600 [1.2800]
High risk sex	18.1 (17.6–18.6)	14.6 (13.3–16.0)	< 0.001 [73.2414]	18.0 (17.5–18.5)	12.1 (10.1–14.4)	< 0.001 [113.7157]	17.9 (17.4–18.4)	15.9 (14.1–17.8)	0.056 [17.3292]

Note. p values are obtained from Chi-2 tests of associations between receipt of social protection and HIV risk factors

In eSwatini, condomless sex and high-risk sex were significantly less frequent among youth in households receiving social transfers and educational support, compared to youth in households without these respective supports (Fig. 1, appendix p. 5). In Malawi, Zambia, and Zimbabwe, condomless sex was significantly less frequent among youth in households with educational support. In Zimbabwe, the prevalence of high-risk sex was significantly lower among youth in households with educational support. In Namibia, condomless sex was significantly lower among youth in households with food support.

Table 3 displays the pooled covariate-adjusted odds ratios (OR) for the associations between receiving social protection and individual HIV risk factors. The complete table with pooled OR for covariates is available in the appendix (p. 6). Compared to youth in households without social transfers, those in households with social transfers had lower odds of reporting condomless sex (OR 0.62, 95% CI 0.54–0.70) and high-risk sex (0.50, 0.44–0.56). A reduction in the odds of condomless sex (0.57, 0.46–0.69) and high-risk sex (0.59, 0.47–0.73) was also observed in youth residing in households with versus without educational support. Youth in households with food support had lower odds of reporting all three HIV risk factors, including condomless sex (0.71, 0.61–0.82), multiple sexual partnerships (0.77, 0.63–0.95), and high-risk sex (0.70, 0.60–0.82), as compared to youth in households without food support.

**Table 3:** Covariate-adjusted associations between three social protection provisions (social transfer, educational support, and food support) and individual HIV risk factors (condomless sex, multiple sexual partnerships, and high-risk sex) in six southern African countries (pooled data)

	Condomless sex		Multiple sexual partnerships		High risk sex	
	Adjusted OR (95% CI)	p value	Adjusted OR (95% CI)	p value	Adjusted OR (95% CI)	p value
Social transfer	0.62 (0.54–0.70)	< 0.001	1.04 (0.89–1.22)	0.617	0.50 (0.44–0.56)	< 0.001
Education support	0.57 (0.46–0.69)	< 0.001	0.94 (0.75–1.19)	0.607	0.59 (0.47–0.73)	< 0.001
Food support	0.71 (0.61–0.82)	< 0.001	0.77 (0.63–0.95)	0.018	0.70 (0.60–0.82)	< 0.001
N	36,181		36,336		36,181	

Note. Models are adjusted for sex, place of residence, age, being in a relationship, education attainment, and country. OR = odds ratios

**Fig. 2:**
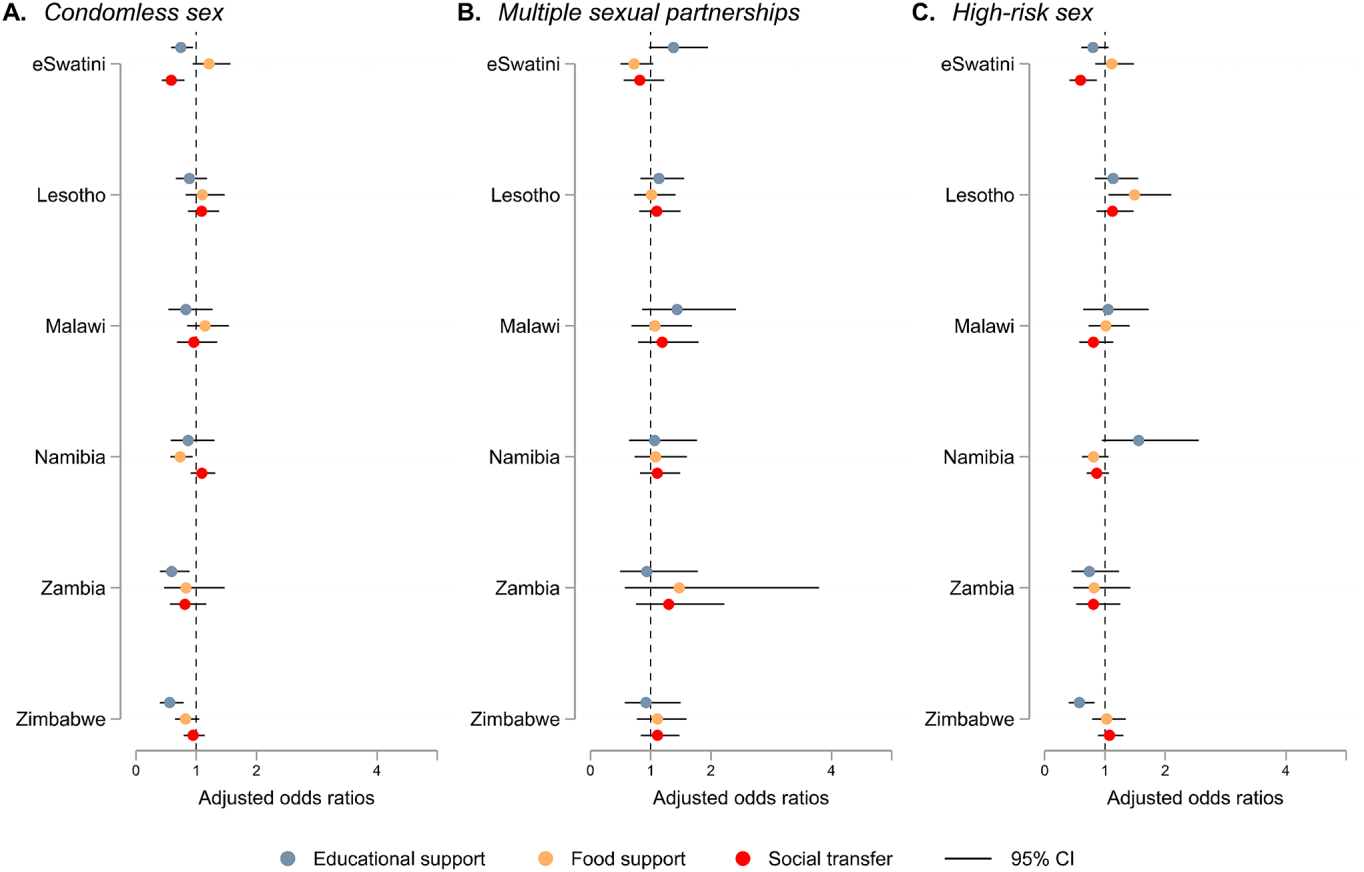
Covariate-adjusted associations between three social protection provisions (social transfer, educational support, and food support) and individual HIV risk factors (condomless sex, multiple sexual partnerships, and high-risk sex) by country Note. Models for each country are adjusted for sex, place of residence, age, being in a relationship, and education attainment. CI = confidence interval. Corresponding data are reported in the appendix (pp. 6–8)

Figure 2 presents the covariate-adjusted odds ratios for the associations of HIV risk factors with receiving social protection in each country (see appendix pp. 6–8 for corresponding data). In eSwatini, Zambia, and Zimbabwe, youth in households with educational support had higher odds of condomless sex compared with youth in households without educational support (eSwatini: OR 0.74, 95% CI 0.58–0.94; Zambia: 0.59, 0.40–0.89; Zimbabwe: 0.56, 0.40–0.79). However, in Zimbabwe, those with educational support had lower odds of high-risk sex (0.57, 0.40–0.82). In Eswatini, receiving social transfers was associated with a reduction in the odds of both condomless sex (0.59, 0.43–0.81) and high-risk sex (0.59, 0.41–0.86). Only in Namibia did youth in households with food support have significantly lower odds of reporting condomless sex (0.73, 0.57–0.94). Conversely, in Lesotho, receiving food support was associated with a significantly increased probability of high-risk sex (1.49, 1.06–2.10).

**Fig. 3:**
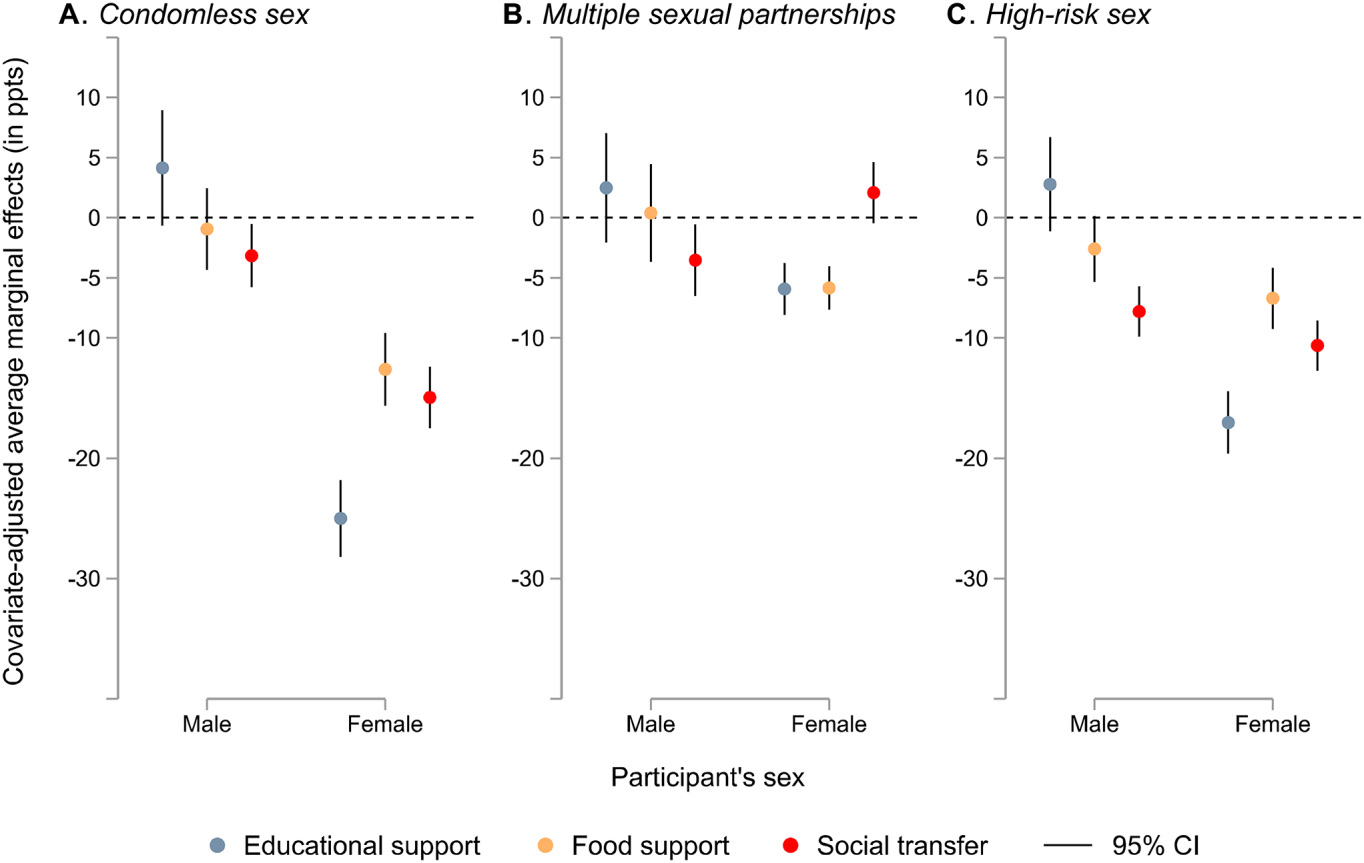
Sex-specific covariate-adjusted associations between three social protection provisions (social transfer, educational support, and food support) and individual HIV risk factors (condomless sex, multiple sexual partnerships, and high-risk sex) in Southern African countries (pooled data) Note. Models are adjusted for sex, place of residence, age, being in a relationship, education attainment, and country. CI = confidence interval, ppts = percentage points. Corresponding data are reported in the appendix (p. 9)

To examine the interaction effect of sex, we conducted analyses controlling for covariates. Figure 3 displays the differences in the probability of reporting HIV risk factors between youth in households with and without social protection (i.e., pooled covariate-adjusted average marginal effects [AME]) by sex (see appendix p. 9 for the pooled data). Our analysis revealed a significant sex interaction effect for all associations except for the association between receiving social transfers and high-risk sex.

Receiving social transfers was associated with a significantly decreased probability of reporting condomless sex for both males (AME − 3.16% points [ppts], 95% CI − 5.79 to − 0.54) and females (–14.94 ppts, − 17.51 to − 12.38). This protective association was significantly stronger for females (appendix p. 9). Social transfers were also associated with a significantly decreased probability of high-risk sex for both males (–7.80 ppts, − 9.88 to − 5.72) and females (–10.62 ppts, − 12.71 to − 8.54), but the protective effect was similar across genders (appendix p. 9).

For females only, receiving educational support was associated with significantly decreased probabilities of condomless sex (–25.01 ppts, − 28.20 to − 21.81), multiple sexual partnerships (–6.93 ppts, − 8.09 to − 3.76), and high-risk sex (–17.03, − 19.62 to − 14.44). Similarly, receiving food support was associated with significantly decreased probabilities of condomless sex (–5.84 ppts, − 7.65 to − 4.03), multiple sexual partnerships (–12.61 ppts, − 15.63 to − 9.59), and high-risk sex (–6.70 ppts, − 9.24 to − 4.15) among females. In contrast, receiving social transfers was associated with a significantly decreased probability of reporting multiple sexual partnerships for males only (–3.53 ppts, − 6.49 to − 0.57).

**Fig. 4:**
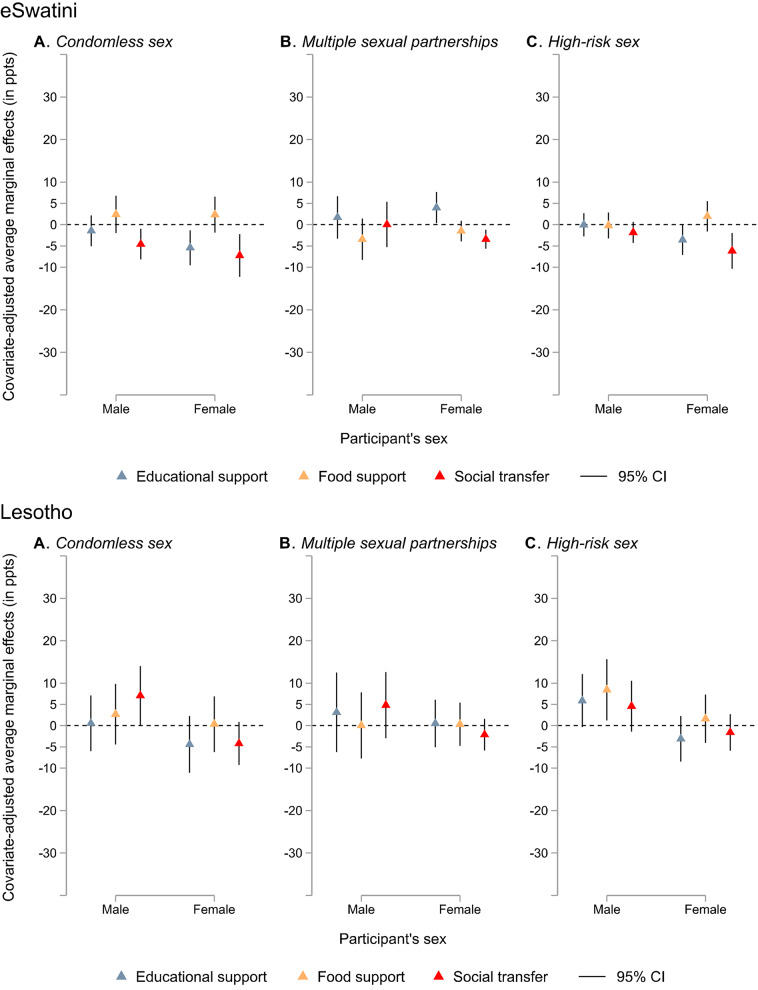

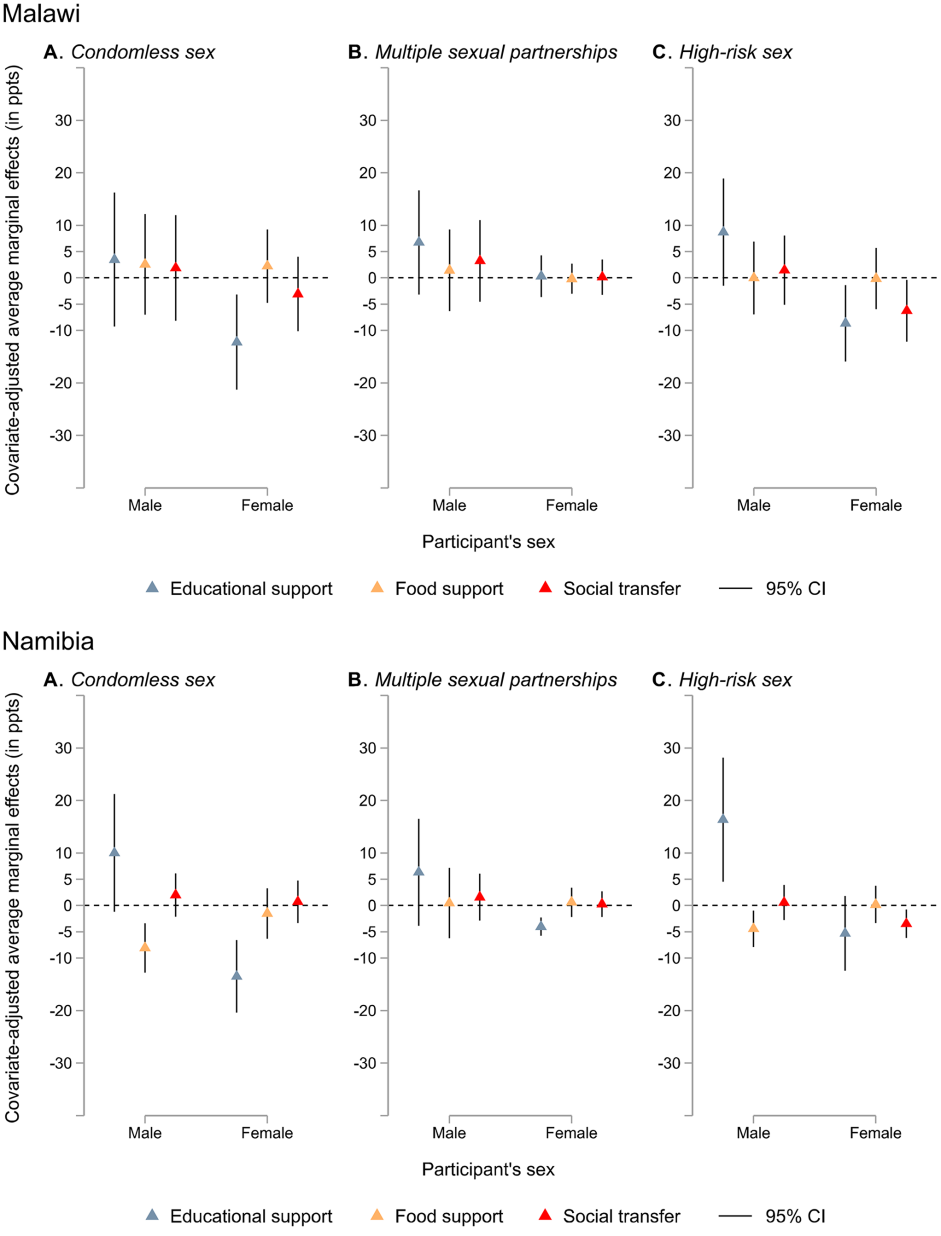

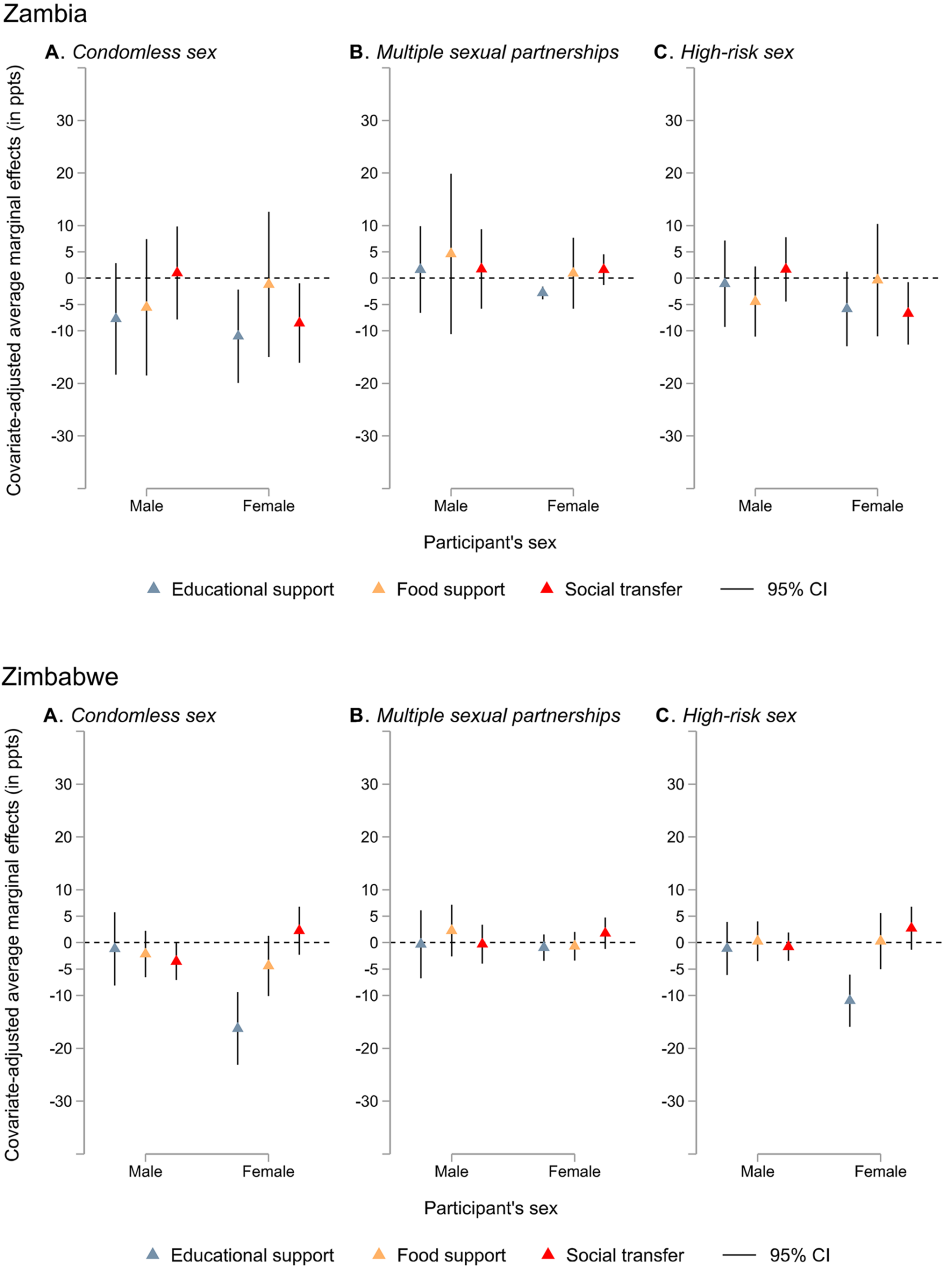
Sex-specific covariate-adjusted associations between three social protection provisions (social transfer, educational support, and food support) and individual HIV risk factors (condomless sex, multiple sexual partnerships, and high-risk sex) by country Note. Models are adjusted for sex, place of residence, age, being in a relationship, education attainment, and country. CI = confidence interval, ppts = percentage points. Corresponding data are reported in the appendix (pp. 9–12)

Country-specific findings are plotted in Fig. 4A-F (see appendix pp. 9–12 for corresponding data). Receiving educational support was associated with a significantly decreased probability of reporting condomless sex for females only in all countries except Lesotho (eSwatini: AME − 5.43 ppts, 95% CI − 9.51 to − 1.34; Malawi: − 12.25 ppts, − 21.30 to − 3.20; Namibia: − 13.49 ppts, − 20.37 to − 6.61; Zambia: − 11.04 ppts, − 19.90 to − 2.17; Zimbabwe: − 16.24 ppts, − 23.09 to − 9.38). Significant sex interaction effects were found on the association between receiving educational support and condomless sex in Namibia and Zimbabwe (appendix pp. 9–12).

Receiving educational support was also linked with significantly decreased probabilities of high-risk sex for females only in eSwatini (–3.55 ppts, − 7.09 to − 0.01), Malawi (–8.64 ppts, − 15.90 to − 1.37), and Zimbabwe (–10.98 ppts, − 15.91 to − 6.04). It was associated with a significantly decreased probability of multiple sexual partnerships for females only in Namibia (–4.04 ppts, − 5.77 to − 2.30) and Zambia (–2.80 ppts, − 3.99 to − 1.60). There was a significant sex interaction effect on the relationship between receiving educational support and high-risk sex in Malawi, Namibia, and Zimbabwe.

Receiving food support, however, was related to significantly decreased probabilities of condomless sex (AME − 8.08 ppts, 95% CI − 12.77 to − 3.38) and high-risk sex (–4.42 ppts, − 7.87 to − 0.97) in boys only in Namibia. A significant sex interaction effect was observed on the association between receiving food support and condomless sex in Namibia.

Receiving social transfers was associated with a significantly decreased probability of condomless sex for both sexes in eSwatini (females: AME − 7.23 ppts, 95% CI − 12.24 to − 2.21; males: − 4.58 ppts, − 8.19 to − 0.97), for females in Zambia (–8.54 ppts, − 16.10 to − 0.98), and for males in Zimbabwe (–3.55 ppts, − 7.04 to − 0.06). It was also associated with significantly decreased probabilities of high-risk sex for females only in eSwatini (–6.16 ppts, − 10.34 to − 1.98), Malawi (–6.26 ppts, − 12.14 to − 0.37), Namibia (–3.48 ppts, − 6.19 to − 0.77), and Zambia (–6.69 ppts, − 12.64 to − 0.75). Additionally, it was associated with a significantly decreased probability of multiple sexual partnerships for females only in eSwatini (–3.42 ppts, − 5.64 to − 1.19). There was a significant sex interaction effect in Zimbabwe regarding the association of receiving social transfers with condomless sex, and in Zambia regarding high-risk sex.

Counter-intuitively, educational support was associated with a significantly increased probability of multiple sexual partnerships among females in eSwatini (4.00 ppts, 0.33 to 7.68) and a significantly increased probability of high-risk sex among males only in Namibia (16.35 ppts, 4.54 to 28.16). In males living in Lesotho, receiving food support was also linked with a significantly increased probability of high-risk sex (8.46 ppts, 1.25 to 15.67). Receiving social transfers was associated with a significantly increased probability of condomless sex (7.07 ppts, 0.09 to 14.05). In addition, there was a significant sex interaction regarding the relationship between receiving social protection and condomless sex in Lesotho.

## Discussion

Our findings underscore the complex relationship between external economic support and HIV risk behaviors among adolescents and youth in Southern Africa. This study highlights the potential of social protection interventions as valuable tools for HIV prevention. Overall, each form of social protection examined (social transfers, educational support, and food support) was independently associated with safer sexual practices among young people. Notably, food support consistently demonstrated an association with decreased odds of engaging in all three HIV risk factor outcomes. In country-specific analyses, educational support played a key role in reducing the likelihood of reporting sexual risk behaviors in three countries (eSwatini, Zambia, and Zimbabwe). Social transfers lowered the odds of reporting high-risk HIV behaviors among young people in eSwatini only. In Namibia, only food support was linked to a decreased likelihood of condomless sex.

These observed reductions in reported HIV risk behaviors among recipients of social protection highlight the potential significance of these programs as pivotal tools in the ongoing fight against HIV. These findings align with a growing body of research investigating the link between social protection programs and their potential to curtail HIV risk behaviors. Although specific outcomes may exhibit variations across studies due to differences in methodologies, target populations, and program structures, numerous studies have consistently identified the role of social protection programs in mitigating HIV-related risks.

For example, a study conducted in South Africa underscores the beneficial impact of cash transfer programs, particularly among young women from economically disadvantaged households [40]. The authors found that cash transfers were associated with a reduced risk of HIV infection, primarily because the improved economic well-being of recipients diminished their vulnerability to engaging in risky sexual behaviors. Several studies conducted in sub-Saharan African countries show that social protection interventions may improve the social and economic well-being of recipients, reducing their vulnerability [41, 42]. Similarly, another study conducted in South Africa emphasized the positive effects of diverse social protection interventions, including financial assistance, psychosocial support, and educational support [5]. These interventions independently contributed to the reduction of HIV risk behaviors among both adolescent boys and girls, further reinforcing the potential of social protection programs to foster healthier choices and diminish susceptibility to HIV infection. By addressing underlying socioeconomic determinants and providing individuals access to essential resources such as food, financial aid, and educational opportunities, social protection programs appear to empower individuals to make informed decisions and proactively reduce their vulnerability to HIV infection.

Our data underscore that social protection measures, particularly those centered on educational support and food assistance, correlate with reduced HIV risk behaviors among beneficiaries. This could be attributed to increased knowledge and awareness fostered by better education, leading to safer sexual practices. Similarly, food assistance might indirectly reduce transactional sex practices, a known risk factor for HIV, by mitigating the pressing need for economic sustenance.

Educational support plays a pivotal role in reducing HIV risk behaviors due to its underlying association with quality schooling and access to educational resources. This contributes to an enhanced understanding of critical aspects such as HIV transmission, preventive measures, and the significance of safer sexual practices. With an improved comprehension of the risks linked to HIV, individuals are better equipped to make informed choices regarding their sexual conduct. Additionally, education empowers individuals, especially adolescents and young adults, by granting them increased autonomy over their lives and prospects. This empowerment materializes through self-confidence, refined negotiation skills, and the capability to assert their sexual health rights. Consequently, individuals are more inclined to engage in safer sexual practices, adeptly navigate peer pressures, and effectively avoid precarious situations.

The mechanism underpinning the reduction of transactional sex practices by food assistance can be attributed to the fundamental human need for sustenance. The consistent provision of food to individuals and households alleviates the economic burdens often associated with risky behaviors, including transactional sex, which is frequently used to secure food or financial support. When this essential need is met through food assistance programs, the incentive for engaging in such risky behavior diminishes significantly [43]. Furthermore, the provision of adequate nutrition extends beyond physical well-being; it also fosters mental and emotional health. Beneficiaries of food assistance experience reduced stress and anxiety typically linked to food insecurity [44]. As a result, they are better positioned to make informed decisions regarding their sexual conduct and actively engage in safer practices.

The link between social transfers and reduced HIV risk behaviors, however, appeared less consistent across the countries studied. We found social transfers beneficial in terms of condom use among young women and men in eSwatini, Zambia, and Zimbabwe. Receiving social transfers in Zimbabwe was associated with a decreased probability of self-reported high-risk sex. This highlights the importance of contextual factors and the potential variations in how cash interventions are implemented. Cash transfers may have a more pronounced effect in countries with substantial economic disparities, where recipients face significant financial challenges. In such contexts, cash transfers can alleviate economic pressures, reducing the need for riskier activities like transactional sex. In addition, cultural norms and attitudes toward HIV and sexual behavior can vary widely between countries, affecting how individuals respond to cash transfer programs in terms of HIV risk reduction. Several studies in the region indicated that social transfer programs are more effective when they are gender-sensitive and have conditionalities related to schooling [9].

Another significant observation from our research is the varying impact of social protection across sexes. Our data suggest that females might benefit more from educational support regarding HIV risk reduction, possibly due to their amplified vulnerabilities, including early marriages, gender-based violence, and societal pressures. Tailoring interventions to consider gender-specific challenges and strengths might optimize the impact of social protection on HIV prevention. Given these gender-specific vulnerabilities and challenges, a one-size-fits-all approach to social protection and HIV prevention may not be as effective. To optimize the impact of social protection programs on HIV prevention, it is essential to tailor interventions to address gender-specific needs, strengths, and challenges.

While our study offers crucial insights, it’s essential to recognize its limitations. Our focus on six Southern African countries, although broad in the regional context, may not encapsulate the full spectrum of social, cultural, and economic diversity within the continent. Future studies should consider expanding the demographic and geographic scope for a more comprehensive understanding. Further, the data are cross-sectional, so inherent limitations, such as establishing a temporal relationship between risk factors and social support, remain. The mechanisms underlying the theories of how social support attenuates HIV risk cannot be fully examined. While the study provides important insights, it is important to acknowledge the inherent challenges in collecting reliable data on sexual behavior, particularly regarding condom use [45]. Self-reported data may be subject to social desirability bias and recall errors, which can influence the interpretation of various sexual risk behaviors (e.g., condomless sex). This study utilizes data from nationally representative surveys, which employ rigorous methodologies to enhance data quality. However, it is crucial to interpret the findings with an awareness of the potential limitations associated with self-reported sexual behavior data. Although care was taken to maintain privacy when noting responses, respondents may be less likely to report HIV-associated behaviors or may provide more socially desirable responses. This survey is designed to be representative of the entire population, but participating youth may not be representative of all youth, including those who chose not to participate. The possibility of residual confounding due to unmeasured or imperfectly measured factors remains.

The implications of these findings are substantial for policymakers, healthcare practitioners, and public health professionals. Expanding investments in social protection initiatives holds the promise of not only enhancing the overall well-being of vulnerable populations but also making significant contributions to the broader effort to reduce HIV transmission rates. Nevertheless, additional research is warranted to delve deeper into the underlying mechanisms through which social protection programs exert their influence on HIV risk factors. Such endeavors will help identify strategies to boost the impact of these programs and bolster our collective response to the HIV/AIDS epidemic.

This study has several strengths. There are few studies investigating the relationship between various forms of social protection and sexual risk behaviors in the region. Using a large, nationally representative sample and inverse probability weighting to reduce selection bias and confounding are also strengths of this study. Contrary to the classical inverse probability technique, the method employed in our study accommodates the study design, yielding robust variances. The study also takes into consideration the fact that the effect of receiving social protection may vary from setting to setting by reporting country-specific estimates. Notwithstanding these strengths, our study has some limitations. First, inverse probability weighting matches those receiving social protection with controls (those not receiving social protection), which leads to better estimates of the effects of social protection. However, estimates presented in this paper rely on the unconfoundedness assumption. Therefore, bias due to unmeasured covariates is not accounted for, which might lead to overestimated effects of receiving social protection on HIV risk behaviors.

In conclusion, social protection interventions appear promising in their potential to reduce HIV risk behaviors among Southern African adolescents and youth. However, a one-size-fits-all approach might not be optimal. Instead, interventions should be context-specific, multifaceted, and responsive to the evolving needs of this age group. Policymakers and stakeholders must invest further in refining these interventions, understanding their limitations, and maximizing their impact in the relentless fight against HIV/AIDS.

## Electronic Supplementary Material

Below is the link to the electronic supplementary material.

Supplementary Material 1

## Declarations

### Data Sharing

All data used in this analysis are deidentified and publicly available at https://phia-data.icap.columbia.edu/datasets. The analytical code is available at https://drive.google.com/drive/folders/13i9Fdhe6SATFMRCPPM4CIbSg7SLY4syP?usp=sharing.
