# Supplementary material for: Social Protection and HIV risk Factors among Youth in Southern Africa: An Analysis of Cross-sectional Population-based HIV Impact Assessment Surveys
Source: AIDS Behav. 2025 Mar 3;29(10):2997–3011. doi: 10.1007/s10461-025-04638-6 (PMC12484328; doi:10.1007/s10461-025-04638-6)
Supplement: Supplementary file 1 — Supplementary Material 1 [file 10461_2025_4638_MOESM1_ESM.docx]

# Supplementary appendix

[Summary of survey design, sampling strategy, and data collection procedures for each country 1](#_Toc179504526)

[Detailed results 4](#_Toc179504527)

## Summary of survey design, sampling strategy, and data collection procedures for each country

**eSwatini**

- **Survey:** SHIMS2 (Swaziland HIV Incidence Measurement Survey)
- **Objective:** Assess HIV-related health indicators
- **Sampling:** Stratified multistage probability sample
- **Sampling Strata:** 8 (four regions by urban/rural status)
- **Sampling Units:**
  - First-stage: Enumeration areas (EAs) selected with probability proportional to the number of households
  - Second-stage: Households within EAs selected from lists compiled by trained staff
  - Final: Eligible individuals within households
- **Precision Goals:**
  - HIV incidence among persons 15-49
  - Viral load suppression (VLS) among HIV+ persons 15-49
  - VLS among HIV+ females 15-24
- **Data Collection:**
  - All adults 15+ years
  - All children 0-14 years in a random half-sample of households
- **Data Collected:**
  - Household interviews
  - Individual interviews
  - Biomarker data (blood samples)

**Malawi**

- **Survey:** MPHIA (Malawi Population-based HIV Impact Assessment)
- **Objective:** Assess HIV-related health indicators
- **Sampling:** Stratified multistage probability sample
- **Sampling Strata:** 7 (five health zones + Lilongwe City + Blantyre City)
- **Sampling Units:**
  - First-stage: Enumeration areas (EAs) selected with probability proportional to the number of households
  - Second-stage: Households within EAs selected from lists compiled by trained staff
  - Final: Eligible individuals within households
- **Precision Goals:**
  - National estimate of HIV incidence
  - Zone-level estimates of viral load suppression (VLS)
- **Data Collection:**
  - All adults 15-64 years
  - All children 0-14 years in a random half-sample of households
- **Data Collected:**
  - Household interviews
  - Individual interviews
  - Biomarker data (blood samples)

**Lesotho**

- **Survey:** LEPHIA (Lesotho Population-based HIV Impact Assessment)
- **Objective:** Assess HIV-related health indicators
- **Sampling:** Stratified multistage probability sample
- **Sampling Strata:** 10 (districts of Lesotho)
- **Sampling Units:**
  - First-stage: Enumeration areas (EAs) selected with probability proportional to the number of households
  - Second-stage: Households within EAs selected from lists compiled by trained staff
  - Final: Eligible individuals within households
- **Precision Goals:**
  - National estimates of HIV incidence
  - District-level estimates of viral load suppression (VLS)
- **Data Collection:**
  - All adults 15-59 years
  - All children 0-14 years in a random half-sample of households
- **Data Collected:**
  - Household interviews
  - Individual interviews
  - Biomarker data (blood samples)

**Namibia**

- **Survey:** NAMPHIA (Namibia Population-based HIV Impact Assessment)
- **Objective:** Assess HIV-related health indicators
- **Sampling:** Stratified multistage probability sample
- **Sampling Strata:** 14 (regions of Namibia)
- **Sampling Units:**
  - First-stage: Enumeration areas (EAs) selected with probability proportional to the number of households
  - Second-stage: Households within EAs selected from lists compiled by trained staff
  - Final: Eligible individuals within households
- **Precision Goals:**
  - National estimates of HIV incidence
  - Regional estimates of viral load suppression (VLS)
- **Data Collection:**
  - Adults 15-64 years
  - Children 0-14 years in a random half-sample of households
- **Data Collected:**
  - Household interviews
  - Individual interviews
  - Biomarker data (blood samples)

**Zambia**

- **Survey:** ZAMPHIA (Zambia Population-based HIV Impact Assessment)
- **Objective:** Assess HIV-related health indicators
- **Sampling:** Stratified multistage probability sample
- **Sampling Strata:** 10 (provinces of Zambia)
- **Sampling Units:**
  - First-stage: Enumeration areas (EAs) selected with probability proportional to the number of households
  - Second-stage: Households within EAs selected from lists compiled by trained staff
  - Final: Eligible individuals within households
- **Precision Goals:**
  - National estimate of HIV incidence
  - Province-level estimates of viral load suppression (VLS)
- **Data Collection:**
  - All adults 15-59 years
  - All children 0-14 years in a random half-sample of households
- **Data Collected:**
  - Household interviews
  - Individual interviews
  - Biomarker data (blood samples)

**Zimbabwe**

- **Survey:** ZIMPHIA (Zimbabwe Population-based HIV Impact Assessment)
- **Objective:** Assess HIV-related health indicators
- **Sampling:** Stratified multistage probability sample
- **Sampling Strata:** 10 (provinces of Zimbabwe)
- **Sampling Units:**
  - First-stage: Enumeration areas (EAs) selected with probability proportional to the number of households
  - Second-stage: Households within EAs selected from lists compiled by trained staff
  - Final: Eligible individuals within households
- **Precision Goals:**
  - National estimate of HIV incidence
  - Province-level estimates of viral load suppression (VLS)
- **Data Collection:**
  - All adults 15+ years
  - All children 0-14 years in a random half-sample of households
- **Data Collected:**
  - Household interviews
  - Individual interviews
  - Biomarker data (blood samples)

## Detailed results

**Table 1. Characteristics of youth aged 15–24 years by country**

|  | **eSwatini (n=3797)** | |  | **Lesotho (n=4420)** | |  | **Malawi (n=7171)** | |  | **Namibia (n=6098)** | |  | **Zambia (n=8089)** | |  | **Zimbabwe (n=7742)** | |
| --- | --- | --- | --- | --- | --- | --- | --- | --- | --- | --- | --- | --- | --- | --- | --- | --- | --- |
|  | n | Weighted % (95% CI) | | n | Weighted % (95% CI) | | n | Weighted % (95% CI) | | n | Weighted % (95% CI) | | n | Weighted % (95% CI) | | n | Weighted % (95% CI) |
| Sex | 3797 |  |  | 4420 |  |  | 7171 |  |  | 6098 |  |  | 8089 |  |  | 7742 |  |
| Male |  | 49.2 (49.2–49.2) | |  | 50.2 (50.2–50.2) | |  | 48.9 (48.9–48.9) | |  | 49.4 (49.4–49.4) | |  | 49.4 (49.4–49.4) | |  | 49.5 (49.5–49.5) |
| Female |  | 50.8 (50.8–50.8) | |  | 49.8 (49.8–49.8) | |  | 51.1 (51.1–51.1) | |  | 50.6 (50.6–50.6) | |  | 50.6 (50.6–50.6) | |  | 50.5 (50.5–50.5) |
| Place of residence | 3797 |  |  | 4420 |  |  | 7171 |  |  | 6098 |  |  | 8089 |  |  | 7742 |  |
| Rural |  | 77.1 (74.3–79.7) | |  | 61.9 (58.9–64.9) | |  | 79.2 (75.9–82.2) | |  | 45.9 (42.9–48.8) | |  | 53.4 (49.7–57.1) | |  | 64.7 (62.9–66.6) |
| Urban |  | 22.9 (20.3–25.7) | |  | 38.1 (35.1–41.1) | |  | 20.8 (17.8–24.1) | |  | 54.1 (51.2–57.1) | |  | 46.6 (42.9–50.3) | |  | 35.3 (33.4–37.1) |
| Participant’s age | 3797 |  |  | 4420 |  |  | 7171 |  |  | 6098 |  |  | 8089 |  |  | 7742 |  |
| 18–24 |  | 69.0 (67.9–70.2) | |  | 67.9 (66.8–69.0) | |  | 67.6 (66.6–68.5) | |  | 71.1 (69.9–72.3) | |  | 69.5 (68.6–70.3) | |  | 65.2 (64.4–66.0) |
| 15–17 |  | 31.0 (29.8–32.1) | |  | 32.1 (31.0–33.2) | |  | 32.4 (31.5–33.4) | |  | 28.9 (27.7–30.1) | |  | 30.5 (29.7–31.4) | |  | 34.8 (34.0–35.6) |
| In a relationship | 3797 | 98.8 (98.3–99.1) | | 4420 | 98.1 (97.7–98.5) | | 7171 | 95.1 (94.5–95.7) | | 6098 | 98.2 (97.8–98.6) | | 8089 | 97.2 (96.8–97.6) | | 7742 | 96.4 (95.9–96.8) |
| Education attainment | 3797 |  |  | 4419 |  |  | 7170 |  |  | 6094 |  |  | 8083 |  |  | 7742 |  |
| None |  | 0.7 (0.5–1.1) |  |  | 1.7 (1.3–2.2) |  |  | 3.7 (3.1–4.3) |  |  | 3.6 (3.0–4.3) |  |  | 2.8 (2.4–3.4) |  |  | 0.6 (0.5–0.8) |
| Primary |  | 60.2 (57.9–62.4) | |  | 28.2 (26.2–30.3) | |  | 61.6 (59.3–63.8) | |  | 88.1 (86.2–89.8) | |  | 35.0 (33.2–37.0) | |  | 19.6 (18.2–21.0) |
| Secondary or higher |  | 39.1 (36.9–41.4) | |  | 70.1 (68.0–72.2) | |  | 34.7 (32.6–37.0) | |  | 8.3 (6.7–10.3) |  |  | 62.1 (60.0–64.2) | |  | 79.8 (78.4–81.2) |
| Any social protection | 3797 | 46.0 (42.8–49.2) | | 4420 | 26.2 (23.8–28.8) | | 7171 | 13.4 (11.9–15.1) | | 6098 | 41.6 (39.4–44.0) | | 8089 | 7.2 (6.1–8.5) |  | 7742 | 21.5 (19.9–23.2) |
| Social transfer | 3797 | 17.8 (15.6–20.3) | | 4420 | 11.7 (10.2–13.3) | | 7171 | 5.5 (4.8–6.4) |  | 6098 | 31.9 (29.8–34.1) | | 8089 | 3.7 (2.9–4.7) |  | 7742 | 13.3 (11.9–14.8) |
| Education support | 3797 | 25.3 (22.0–28.8) | | 4420 | 9.5 (7.7–11.6) |  | 7171 | 3.2 (2.5–4.2) |  | 6098 | 5.4 (4.4–6.8) |  | 8089 | 2.5 (2.0–3.2) |  | 7742 | 5.4 (4.5–6.4) |
| Food support | 3797 | 22.2 (19.9–24.7) | | 4420 | 8.1 (6.7–9.7) |  | 7171 | 6.0 (4.9–7.3) |  | 6098 | 18.3 (16.3–20.5) | | 8089 | 1.7 (1.2–2.3) |  | 7742 | 7.3 (6.3–8.5) |
| Condomless sex | 3720 | 17.0 (15.7–18.4) | | 4367 | 23.9 (22.5–25.4) | | 7047 | 39.9 (38.4–41.4) | | 5714 | 21.8 (20.4–23.1) | | 7773 | 36.1 (34.5–37.7) | | 7614 | 31.5 (30.3–32.7) |
| Multiple sexual partnerships | 3751 | 9.6 (8.5–10.9) |  | 4375 | 18.7 (17.4–20.0) | | 7062 | 10.3 (9.4–11.2) |  | 5720 | 11.7 (10.4–13.1) | | 7835 | 6.7 (6.0–7.4) |  | 7647 | 8.3 (7.6–9.1) |
| High-risk sex | 3720 | 10.8 (9.8–11.9) |  | 4367 | 13.4 (12.4–14.5) | | 7047 | 19.4 (18.3–20.5) | | 5714 | 11.1 (10.1–12.2) | | 7773 | 17.3 (16.4–18.4) | | 7614 | 18.6 (17.7–19.5) |

**Table 2. Comparison of HIV risk factors (condomless sex, multiple sexual partnerships, and high-risk sex) between youth in households with and without each type of social protection (social transfer, educational support, and food support) by country**

|  | Social transfer |  |  |  | Educational support | |  |  | Food support |  |  |
| --- | --- | --- | --- | --- | --- | --- | --- | --- | --- | --- | --- |
|  | No receipt | Receipt | p value | | No receipt | Receipt | p value | | No receipt | Receipt | p value |
| **eSwatini** |  |  |  |  |  |  |  |  |  |  |  |
| Condomless sex | 18.2 (16.7–19.8) | 11.7 (9.2–14.7) | <0.001 |  | 18.1 (16.5–19.8) | 13.7 (11.9–15.9) | 0.002 |  | 16.9 (15.4–18.6) | 17.4 (14.8–20.3) | 0.768 |
| Multiple sexual partnerships | 10.0 (8.8–11.3) | 8.2 (5.9–11.2) | 0.229 |  | 9.7 (8.5–11.1) | 9.4 (7.3–12.0) | 0.780 |  | 10.1 (8.7–11.6) | 8.2 (6.4–10.5) | 0.170 |
| High-risk sex | 11.5 (10.4–12.8) | 7.3 (5.3–9.8) | 0.006 |  | 11.5 (10.3–12.8) | 8.7 (7.1–10.5) | 0.012 |  | 10.8 (9.7–12.0) | 10.7 (8.6–13.2) | 0.917 |
|  |  |  |  |  |  |  |  |  |  |  |  |
| **Lesotho** |  |  |  |  |  |  |  |  |  |  |  |
| Condomless sex | 23.8 (22.4–25.3) | 24.8 (21.0–28.9) | 0.628 |  | 24.3 (22.8–25.9) | 20.2 (16.4–24.6) | 0.076 |  | 23.6 (22.1–25.1) | 27.8 (22.9–33.2) | 0.105 |
| Multiple sexual partnerships | 18.9 (17.6–20.3) | 16.9 (13.2–21.3) | 0.342 |  | 18.7 (17.3–20.1) | 18.9 (14.3–24.6) | 0.927 |  | 18.9 (17.5–20.3) | 16.8 (13.1–21.2) | 0.349 |
| High-risk sex | 13.2 (12.1–14.4) | 14.6 (11.8–18.1) | 0.366 |  | 13.5 (12.4–14.8) | 12.3 (9.6–15.6) | 0.472 |  | 13.1 (12.0–14.2) | 17.3 (13.6–21.8) | 0.034 |
|  |  |  |  |  |  |  |  |  |  |  |  |
| **Malawi** |  |  |  |  |  |  |  |  |  |  |  |
| Condomless sex | 40.1 (38.5–41.7) | 36.7 (30.5–43.5) | 0.335 |  | 40.2 (38.6–41.8) | 29.8 (22.7–38.0) | 0.018 |  | 39.9 (38.3–41.4) | 40.3 (34.0–46.8) | 0.902 |
| Multiple sexual partnerships | 10.2 (9.3–11.2) | 10.8 (7.6–15.2) | 0.739 |  | 10.2 (9.3–11.1) | 13.4 (8.7–20.1) | 0.198 |  | 10.3 (9.4–11.3) | 9.9 (6.9–14.1) | 0.838 |
| High-risk sex | 19.6 (18.5–20.7) | 15.7 (11.9–20.5) | 0.103 |  | 19.5 (18.4–20.7) | 15.9 (10.6–23.2) | 0.296 |  | 19.5 (18.3–20.7) | 18.0 (14.1–22.6) | 0.503 |
|  |  |  |  |  |  |  |  |  |  |  |  |
| **Namibia** |  |  |  |  |  |  |  |  |  |  |  |
| Condomless sex | 22.7 (21.1–24.5) | 19.7 (17.6–22.1) | 0.040 |  | 22.0 (20.6–23.4) | 17.7 (12.6–24.2) | 0.176 |  | 22.7 (21.2–24.3) | 17.7 (14.9–20.9) | 0.010 |
| Multiple sexual partnerships | 12.7 (11.0–14.5) | 9.7 (7.9–11.9) | 0.030 |  | 11.7 (10.4–13.2) | 11.3 (6.9–18.0) | 0.870 |  | 12.3 (10.8–13.9) | 9.2 (6.9–12.2) | 0.066 |
| High-risk sex | 12.0 (10.7–13.4) | 9.2 (7.9–10.7) | 0.007 |  | 11.0 (10.0–12.1) | 12.3 (7.8–18.9) | 0.626 |  | 11.5 (10.4–12.8) | 9.2 (7.5–11.3) | 0.050 |
|  |  |  |  |  |  |  |  |  |  |  |  |
| **Zambia** |  |  |  |  |  |  |  |  |  |  |  |
| Condomless sex | 36.2 (34.6–37.8) | 32.4 (25.6–40.1) | 0.309 |  | 36.4 (34.8–38.0) | 23.3 (17.2–30.9) | 0.002 |  | 36.1 (34.5–37.7) | 35.1 (24.6–47.2) | 0.859 |
| Multiple sexual partnerships | 6.6 (5.9–7.4) | 8.6 (5.4–13.4) | 0.264 |  | 6.6 (6.0–7.4) | 8.7 (4.8–15.5) | 0.354 |  | 6.7 (6.0–7.4) | 8.9 (4.0–18.3) | 0.447 |
| High-risk sex | 17.5 (16.5–18.5) | 14.2 (10.2–19.5) | 0.205 |  | 17.5 (16.5–18.5) | 13.0 (8.3–19.7) | 0.154 |  | 17.4 (16.5–18.4) | 13.8 (8.4–21.9) | 0.305 |
|  |  |  |  |  |  |  |  |  |  |  |  |
| **Zimbabwe** |  |  |  |  |  |  |  |  |  |  |  |
| Condomless sex | 31.9 (30.5–33.3) | 28.9 (26.1–31.9) | 0.086 |  | 32.3 (31.0–33.7) | 17.6 (13.5–22.6) | <0.001 |  | 31.7 (30.5–33.1) | 28.5 (24.3–33.1) | 0.172 |
| Multiple sexual partnerships | 8.3 (7.5–9.1) | 8.3 (6.6–10.4) | 0.977 |  | 8.4 (7.7–9.2) | 6.9 (4.5–10.4) | 0.347 |  | 8.3 (7.6–9.1) | 8.3 (6.1–11.2) | 0.995 |
| High-risk sex | 18.9 (17.9–19.9) | 16.9 (14.8–19.3) | 0.136 |  | 19.1 (18.1–20.1) | 10.2 (7.6–13.7) | <0.001 |  | 18.6 (17.7–19.6) | 18.1 (14.9–21.8) | 0.773 |

**Table 3. Covariate–adjusted associations between three social protection provisions (social transfer, educational support, and food support) and individual HIV risk factors (condomless sex, multiple sexual partnerships, and high-risk sex) in six Southern African countries (pooled data)**

|  | Condomless sex |  |  | Multiple sexual partnerships | |  | High-risk sex |  |
| --- | --- | --- | --- | --- | --- | --- | --- | --- |
|  | Adjusted OR (95% CI) | p value |  | Adjusted OR (95% CI) | p value |  | Adjusted OR (95% CI) | p value |
| Social transfer | 0.62 (0.54–0.70) | <0.001 |  | 1.04 (0.89–1.22) | 0.617 |  | 0.50 (0.44–0.56) | <0.001 |
| Education support | 0.57 (0.46–0.69) | <0.001 |  | 0.94 (0.75–1.19) | 0.607 |  | 0.59 (0.47–0.73) | <0.001 |
| Food support | 0.71 (0.61–0.82) | <0.001 |  | 0.77 (0.63–0.95) | 0.018 |  | 0.70 (0.60–0.82) | <0.001 |
| Female vs. male | 2.18 (2.02–2.35) | <0.001 |  | 0.29 (0.25–0.32) | <0.001 |  | 2.15 (1.96–2.36) | <0.001 |
| Urban residence vs. rural residence | 0.88 (0.80–0.96) | 0.005 |  | 1.67 (1.46–1.91) | <0.001 |  | 0.96 (0.88–1.05) | 0.339 |
| Participant 15–17 vs. 18–24 | 0.09 (0.09–0.10) | <0.001 |  | 0.21 (0.17–0.25) | <0.001 |  | 0.09 (0.08–0.10) | <0.001 |
| In a relationship | 0.17 (0.14–0.20) | <0.001 |  | 0.43 (0.35–0.52) | <0.001 |  | 0.16 (0.14–0.19) | <0.001 |
| Education attainment | |  |  |  |  |  |  |  |
| No education | 1 | ·· |  | 1 | ·· |  | 1 | ·· |
| Primary | 0.93 (0.75–1.16) | 0.506 |  | 0.50 (0.34–0.75) | 0.002 |  | 0.52 (0.40–0.67) | <0.001 |
| Secondary or higher | 0.70 (0.56–0.88) | 0.004 |  | 0.75 (0.50–1.12) | 0.151 |  | 0.54 (0.42–0.70) | <0.001 |
| Country |  |  |  |  |  |  |  |  |
| eSwatini | 1 | ·· |  | 1 | ·· |  | 1 | ·· |
| Lesotho | 4.25 (3.72–4.86) | <0.001 |  | 2.16 (1.80–2.60) | <0.001 |  | 2.40 (2.05–2.80) | <0.001 |
| Malawi | 4.98 (4.37–5.67) | <0.001 |  | 1.46 (1.21–1.76) | <0.001 |  | 2.88 (2.48–3.33) | <0.001 |
| Namibia | 3.99 (3.46–4.60) | <0.001 |  | 1.91 (1.53–2.39) | <0.001 |  | 2.78 (2.37–3.26) | <0.001 |
| Zambia | 2.75 (2.38–3.18) | <0.001 |  | 0.39 (0.32–0.48) | <0.001 |  | 1.72 (1.47–2.01) | <0.001 |
| Zimbabwe | 3.94 (3.43–4.53) | <0.001 |  | 0.74 (0.61–0.90) | 0.004 |  | 2.52 (2.18–2.91) | <0.001 |
| N | 36181 |  |  | 36336 |  |  | 36181 |  |

**Table 4. Covariate–adjusted associations between three social protection provisions (social transfer, educational support, and food support) and individual HIV risk factors (condomless sex, multiple sexual partnerships, and high-risk sex) by country**

1. **eSwatini**

|  | Condomless sex |  |  | Multiple sexual partnerships | |  | High-risk sex |  |
| --- | --- | --- | --- | --- | --- | --- | --- | --- |
|  | Adjusted OR (95% CI) | p value |  | Adjusted OR (95% CI) | p value |  | Adjusted OR (95% CI) | p value |
| Social transfer | 0.59 (0.43–0.81) | 0.002 |  | 0.82 (0.55–1.22) | 0.319 |  | 0.59 (0.41–0.86) | 0.008 |
| Education support | 0.74 (0.58–0.94) | 0.017 |  | 1.38 (0.97–1.95) | 0.068 |  | 0.80 (0.60–1.06) | 0.115 |
| Food support | 1.21 (0.94–1.57) | 0.133 |  | 0.73 (0.50–1.04) | 0.081 |  | 1.11 (0.84–1.48) | 0.447 |
| Female vs. male | 2.35 (1.86–2.97) | <0.001 |  | 0.34 (0.26–0.44) | <0.001 |  | 3.27 (2.50–4.29) | <0.001 |
| Urban residence vs. rural residence | 1.04 (0.81–1.34) | 0.748 |  | 1.13 (0.77–1.67) | 0.514 |  | 0.94 (0.73–1.21) | 0.614 |
| Participant 15–17 vs. 18–24 | 0.10 (0.07–0.14) | <0.001 |  | 0.13 (0.08–0.21) | <0.001 |  | 0.13 (0.08–0.20) | <0.001 |
| In a relationship | 0.22 (0.10–0.49) | <0.001 |  | 0.12 (0.05–0.26) | <0.001 |  | 0.18 (0.08–0.37) | <0.001 |
| Education attainment | |  |  |  |  |  |  |  |
| No education | 1 | ·· |  | 1 | ·· |  | 1 | ·· |
| Primary | 0.46 (0.14–1.48) | 0.185 |  | 0.75 (0.22–2.63) | 0.646 |  | 1.02 (0.28–3.75) | 0.977 |
| Secondary or higher | 0.28 (0.09–0.91) | 0.035 |  | 0.89 (0.25–3.13) | 0.852 |  | 0.70 (0.19–2.58) | 0.576 |
| N | 3718 |  |  | 3749 |  |  | 3718 |  |

1. **Lesotho**

|  | Condomless sex |  |  | Multiple sexual partnerships | |  | High-risk sex |  |
| --- | --- | --- | --- | --- | --- | --- | --- | --- |
|  | Adjusted OR (95% CI) | p value |  | Adjusted OR (95% CI) | p value |  | Adjusted OR (95% CI) | p value |
| Social transfer | 1.09 (0.86–1.38) | 0.467 |  | 1.10 (0.81–1.49) | 0.522 |  | 1.12 (0.86–1.47) | 0.383 |
| Education support | 0.89 (0.66–1.18) | 0.395 |  | 1.14 (0.83–1.56) | 0.404 |  | 1.13 (0.83–1.55) | 0.409 |
| Food support | 1.10 (0.82–1.47) | 0.499 |  | 1.01 (0.73–1.41) | 0.927 |  | 1.49 (1.06–2.10) | 0.025 |
| Female vs. male | 1.60 (1.37–1.88) | <0.001 |  | 0.21 (0.17–0.26) | <0.001 |  | 1.27 (1.04–1.57) | 0.023 |
| Urban residence vs. rural residence | 0.89 (0.76–1.05) | 0.160 |  | 1.37 (1.13–1.66) | 0.002 |  | 1.10 (0.90–1.34) | 0.336 |
| Participant 15–17 vs. 18–24 | 0.16 (0.13–0.20) | <0.001 |  | 0.25 (0.20–0.32) | <0.001 |  | 0.18 (0.13–0.24) | <0.001 |
| In a relationship | 0.70 (0.43–1.14) | 0.148 |  | 0.26 (0.15–0.46) | <0.001 |  | 0.53 (0.31–0.90) | 0.020 |
| Education attainment | |  |  |  |  |  |  |  |
| No education | 1 | ·· |  | 1 | ·· |  | 1 | ·· |
| Primary | 1.23 (0.70–2.15) | 0.455 |  | 0.57 (0.32–1.01) | 0.053 |  | 1.05 (0.50–2.20) | 0.885 |
| Secondary or higher | 0.81 (0.47–1.38) | 0.424 |  | 0.65 (0.37–1.16) | 0.142 |  | 0.75 (0.37–1.51) | 0.405 |
| N | 4351 |  |  | 4359 |  |  | 4351 |  |

1. **Malawi**

|  | Condomless sex |  |  | Multiple sexual partnerships | |  | High-risk sex |  |
| --- | --- | --- | --- | --- | --- | --- | --- | --- |
|  | Adjusted OR (95% CI) | p value |  | Adjusted OR (95% CI) | p value |  | Adjusted OR (95% CI) | p value |
| Social transfer | 0.96 (0.68–1.35) | 0.806 |  | 1.19 (0.79–1.79) | 0.382 |  | 0.81 (0.58–1.13) | 0.206 |
| Education support | 0.83 (0.54–1.27) | 0.371 |  | 1.44 (0.86–2.41) | 0.160 |  | 1.05 (0.64–1.73) | 0.836 |
| Food support | 1.15 (0.85–1.54) | 0.356 |  | 1.07 (0.68–1.68) | 0.750 |  | 1.01 (0.73–1.41) | 0.930 |
| Female vs. male | 1.70 (1.47–1.96) | <0.001 |  | 0.18 (0.14–0.22) | <0.001 |  | 1.96 (1.66–2.32) | <0.001 |
| Urban residence vs. rural residence | 0.89 (0.76–1.05) | 0.164 |  | 1.46 (1.13–1.88) | 0.006 |  | 1.14 (0.96–1.35) | 0.133 |
| Participant 15–17 vs. 18–24 | 0.11 (0.09–0.14) | <0.001 |  | 0.31 (0.22–0.42) | <0.001 |  | 0.18 (0.13–0.24) | <0.001 |
| In a relationship | 0.83 (0.63–1.08) | 0.160 |  | 0.47 (0.33–0.67) | <0.001 |  | 0.60 (0.45–0.80) | 0.001 |
| Education attainment | |  |  |  |  |  |  |  |
| No education | 1 | ·· |  | 1 | ·· |  | 1 | ·· |
| Primary | 0.68 (0.48–0.97) | 0.036 |  | 0.85 (0.45–1.63) | 0.617 |  | 0.49 (0.33–0.73) | 0.001 |
| Secondary or higher | 0.31 (0.21–0.45) | <0.001 |  | 0.70 (0.36–1.36) | 0.282 |  | 0.31 (0.21–0.47) | <0.001 |
| N | 7046 |  |  | 7061 |  |  | 7046 |  |

1. **Namibia**

|  | Condomless sex |  |  | Multiple sexual partnerships | |  | High-risk sex |  |
| --- | --- | --- | --- | --- | --- | --- | --- | --- |
|  | Adjusted OR (95% CI) | p value |  | Adjusted OR (95% CI) | p value |  | Adjusted OR (95% CI) | p value |
| Social transfer | 1.09 (0.91–1.31) | 0.332 |  | 1.11 (0.83–1.49) | 0.471 |  | 0.86 (0.70–1.07) | 0.164 |
| Education support | 0.87 (0.58–1.30) | 0.472 |  | 1.07 (0.64–1.77) | 0.794 |  | 1.56 (0.95–2.55) | 0.076 |
| Food support | 0.73 (0.57–0.94) | 0.015 |  | 1.08 (0.73–1.60) | 0.681 |  | 0.81 (0.62–1.06) | 0.115 |
| Female vs. male | 1.53 (1.29–1.82) | <0.001 |  | 0.19 (0.14–0.24) | <0.001 |  | 1.53 (1.21–1.92) | <0.001 |
| Urban residence vs. rural residence | 0.97 (0.81–1.16) | 0.707 |  | 2.08 (1.55–2.80) | <0.001 |  | 1.03 (0.81–1.30) | 0.803 |
| Participant 15–17 vs. 18–24 | 0.23 (0.19–0.30) | <0.001 |  | 0.13 (0.08–0.20) | <0.001 |  | 0.23 (0.15–0.33) | <0.001 |
| In a relationship | 0.16 (0.10–0.26) | <0.001 |  | 0.77 (0.42–1.39) | 0.371 |  | 0.21 (0.12–0.38) | <0.001 |
| Education attainment | |  |  |  |  |  |  |  |
| No education | 1 | ·· |  | 1 | ·· |  | 1 | ·· |
| Primary | 0.45 (0.30–0.66) | <0.001 |  | 0.89 (0.46–1.72) | 0.722 |  | 0.57 (0.33–1.00) | 0.052 |
| Secondary or higher | 0.49 (0.29–0.84) | 0.011 |  | 1.65 (0.72–3.75) | 0.223 |  | 0.67 (0.32–1.39) | 0.265 |
| N | 5710 |  |  | 5716 |  |  | 5710 |  |

1. **Zambia**

|  | Condomless sex |  |  | Multiple sexual partnerships | |  | High-risk sex |  |
| --- | --- | --- | --- | --- | --- | --- | --- | --- |
|  | Adjusted OR (95% CI) | p value |  | Adjusted OR (95% CI) | p value |  | Adjusted OR (95% CI) | p value |
| Social transfer | 0.81 (0.57–1.17) | 0.251 |  | 1.30 (0.76–2.22) | 0.323 |  | 0.81 (0.52–1.25) | 0.324 |
| Education support | 0.59 (0.40–0.89) | 0.013 |  | 0.94 (0.49–1.78) | 0.836 |  | 0.74 (0.44–1.24) | 0.239 |
| Food support | 0.83 (0.47–1.47) | 0.510 |  | 1.47 (0.57–3.80) | 0.406 |  | 0.82 (0.47–1.42) | 0.462 |
| Female vs. male | 1.60 (1.39–1.85) | <0.001 |  | 0.16 (0.13–0.22) | <0.001 |  | 2.69 (2.27–3.19) | <0.001 |
| Urban residence vs. rural residence | 0.60 (0.50–0.72) | <0.001 |  | 0.63 (0.48–0.83) | 0.002 |  | 0.72 (0.60–0.86) | <0.001 |
| Participant 15–17 vs. 18–24 | 0.14 (0.11–0.16) | <0.001 |  | 0.32 (0.24–0.44) | <0.001 |  | 0.18 (0.15–0.23) | <0.001 |
| In a relationship | 0.54 (0.40–0.72) | <0.001 |  | 0.18 (0.11–0.28) | <0.001 |  | 0.36 (0.26–0.49) | <0.001 |
| Education attainment | |  |  |  |  |  |  |  |
| No education | 1 | ·· |  | 1 | ·· |  | 1 | ·· |
| Primary | 1.22 (0.84–1.78) | 0.287 |  | 1.05 (0.43–2.55) | 0.911 |  | 1.73 (1.18–2.54) | 0.007 |
| Secondary or higher | 0.50 (0.34–0.74) | 0.001 |  | 1.45 (0.60–3.51) | 0.389 |  | 1.10 (0.75–1.62) | 0.600 |
| N | 7742 |  |  | 7804 |  |  | 7742 |  |

1. **Zimbabwe**

|  | Condomless sex |  |  | Multiple sexual partnerships | |  | High-risk sex |  |
| --- | --- | --- | --- | --- | --- | --- | --- | --- |
|  | Adjusted OR (95% CI) | p value |  | Adjusted OR (95% CI) | p value |  | Adjusted OR (95% CI) | p value |
| Social transfer | 0.95 (0.79–1.14) | 0.558 |  | 1.11 (0.84–1.48) | 0.444 |  | 1.07 (0.88–1.30) | 0.454 |
| Education support | 0.56 (0.40–0.79) | 0.002 |  | 0.92 (0.57–1.50) | 0.739 |  | 0.57 (0.40–0.82) | 0.004 |
| Food support | 0.82 (0.65–1.05) | 0.109 |  | 1.11 (0.78–1.60) | 0.547 |  | 1.03 (0.79–1.34) | 0.829 |
| Female vs. male | 3.54 (3.10–4.05) | <0.001 |  | 0.20 (0.15–0.25) | <0.001 |  | 4.72 (3.92–5.68) | <0.001 |
| Urban residence vs. rural residence | 0.70 (0.60–0.82) | <0.001 |  | 1.61 (1.26–2.06) | <0.001 |  | 0.84 (0.71–0.98) | 0.030 |
| Participant 15–17 vs. 18–24 | 0.09 (0.08–0.11) | <0.001 |  | 0.18 (0.13–0.26) | <0.001 |  | 0.11 (0.09–0.13) | <0.001 |
| In a relationship | 0.56 (0.42–0.76) | <0.001 |  | 0.21 (0.14–0.31) | <0.001 |  | 0.47 (0.35–0.64) | <0.001 |
| Education attainment | |  |  |  |  |  |  |  |
| No education | 1 | ·· |  | 1 | ·· |  | 1 | ·· |
| Primary | 1.09 (0.56–2.14) | 0.787 |  | 0.56 (0.18–1.75) | 0.300 |  | 0.87 (0.39–1.95) | 0.732 |
| Secondary or higher | 0.55 (0.28–1.06) | 0.073 |  | 0.45 (0.15–1.42) | 0.167 |  | 0.51 (0.23–1.15) | 0.102 |
| N | 7614 |  |  | 7647 |  |  | 7614 |  |

**Table 5. Sex–specific covariate–adjusted associations between three social protection provisions (social transfer, educational support, and food support) and individual HIV risk factors (condomless sex, multiple sexual partnerships, and high-risk sex) in Southern African countries (pooled data)**

|  | Condomless sex |  |  | Multiple sexual partnerships | |  | High-risk sex |  |
| --- | --- | --- | --- | --- | --- | --- | --- | --- |
|  | AME (95% CI) | p value |  | AME (95% CI) | p value | | AME (95% CI) | p value |
| *Social transfer* |  |  |  |  |  |  |  |  |
| For males | –0.0316 (–0.0579 to –0.0054) | 0.020 |  | –0.0353 (–0.0649 to –0.0057) | 0.021 | | –0.0780 (–0.0988 to –0.0572) | <0.001 |
| For females | –0.1494 (–0.1751 to –0.1238) | <0.001 |  | 0.0209 (–0.0046 to 0.0463) | 0.104 | | –0.1062 (–0.1271 to –0.0854) | <0.001 |
| Second difference (Reference: males) | –0.1178 (–0.1511 to –0.0846) | <0.001 |  | 0.0562 (0.0158 to 0.0966) | 0.008 | | –0.0282 (–0.0574 to 0.0009) | 0.057 |
| *Educational support* |  |  |  |  |  |  |  |  |
| For males | 0.0414 (–0.0066 to 0.0893) | 0.088 |  | 0.0248 (–0.0205 to 0.0702) | 0.271 | | 0.0278 (–0.0114 to 0.0669) | 0.156 |
| For females | –0.2501 (–0.2820 to –0.2181) | <0.001 |  | –0.0593 (–0.0809 to –0.0376) | <0.001 | | –0.1703 (–0.1962 to –0.1444) | <0.001 |
| Second difference (Reference: males) | –0.2914 (–0.3472 to –0.2356) | <0.001 |  | –0.0841 (–0.1355 to –0.0327) | 0.002 | | –0.1981 (–0.2440 to –0.1523) | <0.001 |
| *Food support* |  |  |  |  |  |  |  |  |
| For males | –0.0094 (–0.0433 to 0.0244) | 0.571 |  | 0.0040 (–0.0366 to 0.0445) | 0.842 | | –0.0260 (–0.0532 to 0.0013) | 0.061 |
| For females | –0.1261 (–0.1563 to –0.0959) | <0.001 |  | –0.0584 (–0.0765 to –0.0403) | <0.001 | | –0.0670 (–0.0924 to –0.0415) | <0.001 |
| Second difference (Reference: males) | –0.1167 (–0.1629 to –0.0704) | <0.001 |  | –0.0623 (–0.1079 to –0.0168) | 0.009 | | –0.0410 (–0.0789 to –0.0030) | 0.035 |

**Table 6. Sex–specific covariate–adjusted associations between three social protection provisions (social transfer, educational support, and food support) and individual HIV risk factors (condomless sex, multiple sexual partnerships, and high-risk sex) by country**

1. **eSwatini**

|  | Condomless sex |  |  | Multiple sexual partnerships | |  | High-risk sex |  |
| --- | --- | --- | --- | --- | --- | --- | --- | --- |
|  | AME (95% CI) | p value |  | AME (95% CI) | p value |  | AME (95% CI) | p value |
| *Social transfer* |  |  |  |  |  |  |  |  |
| For males | –0.0458 (–0.0819 to –0.0097) | 0.015 |  | 0.0005 (–0.0528 to 0.0538) | 0.984 |  | –0.0182 (–0.0429 to 0.0066) | 0.143 |
| For females | –0.0723 (–0.1224 to –0.0221) | 0.007 |  | –0.0342 (–0.0564 to –0.0119) | 0.004 |  | –0.0616 (–0.1034 to –0.0198) | 0.006 |
| Second difference (Reference: males) | –0.0265 (–0.0868 to 0.0339) | 0.375 |  | –0.0347 (–0.0908 to 0.0215) | 0.215 |  | –0.0434 (–0.0904 to 0.0036) | 0.069 |
| *Educational support* | |  |  |  |  |  |  |  |
| For males | –0.0143 (–0.0504 to 0.0217) | 0.421 |  | 0.0172 (–0.0327 to 0.0671) | 0.485 |  | –0.0003 (–0.0275 to 0.0270) | 0.985 |
| For females | –0.0543 (–0.0951 to –0.0134) | 0.011 |  | 0.0400 (0.0033 to 0.0768) | 0.034 |  | –0.0355 (–0.0709 to –0.0001) | 0.049 |
| Second difference (Reference: males) | –0.0399 (–0.0954 to 0.0155) | 0.150 |  | 0.0229 (–0.0344 to 0.0801) | 0.419 |  | –0.0353 (–0.0795 to 0.0090) | 0.113 |
| *Food support* |  |  |  |  |  |  |  |  |
| For males | 0.0242 (–0.0195 to 0.0678) | 0.265 |  | –0.0342 (–0.0828 to 0.0144) | 0.160 |  | –0.0020 (–0.0324 to 0.0283) | 0.892 |
| For females | 0.0236 (–0.0187 to 0.0660) | 0.261 |  | –0.0148 (–0.0390 to 0.0094) | 0.219 |  | 0.0198 (–0.0158 to 0.0554) | 0.263 |
| Second difference (Reference: males) | –0.0005 (–0.0561 to 0.0550) | 0.984 |  | 0.0194 (–0.0345 to 0.0732) | 0.466 |  | 0.0218 (–0.0210 to 0.0647) | 0.304 |

1. **Lesotho**

|  | Condomless sex |  |  | Multiple sexual partnerships | |  | High-risk sex |  |
| --- | --- | --- | --- | --- | --- | --- | --- | --- |
|  | AME (95% CI) | p value |  | AME (95% CI) | p value |  | AME (95% CI) | p value |
| *Social transfer* |  |  |  |  |  |  |  |  |
| For males | 0.0707 (0.0009 to 0.1405) | 0.047 |  | 0.0483 (–0.0295 to 0.1261) | 0.213 |  | 0.0457 (–0.0142 to 0.1056) | 0.128 |
| For females | –0.0419 (–0.0927 to 0.0088) | 0.101 |  | –0.0211 (–0.0583 to 0.0161) | 0.253 |  | –0.0159 (–0.0588 to 0.0270) | 0.453 |
| Second difference (Reference: males) | –0.1127 (–0.2017 to –0.0237) | 0.015 |  | –0.0694 (–0.1573 to 0.0184) | 0.116 |  | –0.0616 (–0.1398 to 0.0165) | 0.117 |
| *Educational support* | |  |  |  |  |  |  |  |
| For males | 0.0056 (–0.0601 to 0.0713) | 0.862 |  | 0.0314 (–0.0624 to 0.1253) | 0.497 |  | 0.0590 (–0.0036 to 0.1216) | 0.064 |
| For females | –0.0441 (–0.1111 to 0.0230) | 0.188 |  | 0.0052 (–0.0508 to 0.0613) | 0.849 |  | –0.0309 (–0.0847 to 0.0229) | 0.248 |
| Second difference (Reference: males) | –0.0497 (–0.1431 to 0.0437) | 0.284 |  | –0.0262 (–0.1527 to 0.1003) | 0.674 |  | –0.0899 (–0.1798 to –0.0000) | 0.050 |
| *Food support* |  |  |  |  |  |  |  |  |
| For males | 0.0270 (–0.0441 to 0.0981) | 0.442 |  | 0.0005 (–0.0774 to 0.0784) | 0.990 |  | 0.0846 (0.0125 to 0.1567) | 0.023 |
| For females | 0.0035 (–0.0619 to 0.0689) | 0.914 |  | 0.0034 (–0.0476 to 0.0544) | 0.891 |  | 0.0163 (–0.0406 to 0.0733) | 0.560 |
| Second difference (Reference: males) | –0.0235 (–0.1168 to 0.0698) | 0.608 |  | 0.0029 (–0.0925 to 0.0984) | 0.950 |  | –0.0683 (–0.1566 to 0.0201) | 0.124 |

1. **Malawi**

|  | Condomless sex |  |  | Multiple sexual partnerships | |  | High-risk sex |  |
| --- | --- | --- | --- | --- | --- | --- | --- | --- |
|  | AME (95% CI) | p value |  | AME (95% CI) | p value |  | AME (95% CI) | p value |
| *Social transfer* |  |  |  |  |  |  |  |  |
| For males | 0.0189 (–0.0816 to 0.1193) | 0.702 |  | 0.0323 (–0.0455 to 0.1101) | 0.400 |  | 0.0146 (–0.0512 to 0.0805) | 0.651 |
| For females | –0.0308 (–0.1017 to 0.0400) | 0.379 |  | 0.0014 (–0.0322 to 0.0351) | 0.931 |  | –0.0626 (–0.1214 to –0.0037) | 0.038 |
| Second difference (Reference: males) | –0.0497 (–0.1631 to 0.0637) | 0.375 |  | –0.0309 (–0.1185 to 0.0567) | 0.474 |  | –0.0772 (–0.1693 to 0.0149) | 0.097 |
| *Educational support* | |  |  |  |  |  |  |  |
| For males | 0.0349 (–0.0926 to 0.1623) | 0.578 |  | 0.0676 (–0.0315 to 0.1667) | 0.173 |  | 0.0870 (–0.0151 to 0.1891) | 0.092 |
| For females | –0.1225 (–0.2130 to –0.0320) | 0.010 |  | 0.0030 (–0.0366 to 0.0426) | 0.877 |  | –0.0864 (–0.1590 to –0.0137) | 0.022 |
| Second difference (Reference: males) | –0.1574 (–0.3165 to 0.0017) | 0.052 |  | –0.0646 (–0.1687 to 0.0396) | 0.213 |  | –0.1733 (–0.2958 to –0.0509) | 0.007 |
| *Food support* |  |  |  |  |  |  |  |  |
| For males | 0.0257 (–0.0700 to 0.1214) | 0.585 |  | 0.0143 (–0.0633 to 0.0919) | 0.707 |  | –0.0001 (–0.0694 to 0.0691) | 0.997 |
| For females | 0.0224 (–0.0475 to 0.0923) | 0.515 |  | –0.0018 (–0.0305 to 0.0268) | 0.896 |  | –0.0015 (–0.0597 to 0.0567) | 0.958 |
| Second difference (Reference: males) | –0.0033 (–0.1239 to 0.1174) | 0.956 |  | –0.0162 (–0.0967 to 0.0644) | 0.683 |  | –0.0014 (–0.0908 to 0.0881) | 0.975 |

1. **Namibia**

|  | Condomless sex |  |  | Multiple sexual partnerships | |  | High-risk sex |  |
| --- | --- | --- | --- | --- | --- | --- | --- | --- |
|  | AME (95% CI) | p value |  | AME (95% CI) | p value |  | AME (95% CI) | p value |
| *Social transfer* |  |  |  |  |  |  |  |  |
| For males | 0.0201 (–0.0211 to 0.0614) | 0.324 |  | 0.0158 (–0.0287 to 0.0604) | 0.470 |  | 0.0058 (–0.0274 to 0.0390) | 0.722 |
| For females | 0.0071 (–0.0331 to 0.0474) | 0.718 |  | 0.0026 (–0.0217 to 0.0269) | 0.824 |  | –0.0348 (–0.0619 to –0.0077) | 0.014 |
| Second difference (Reference: males) | –0.0130 (–0.0690 to 0.0430) | 0.636 |  | –0.0132 (–0.0587 to 0.0323) | 0.555 |  | –0.0406 (–0.0853 to 0.0041) | 0.073 |
| *Educational support* | |  |  |  |  |  |  |  |
| For males | 0.1002 (–0.0117 to 0.2120) | 0.077 |  | 0.0633 (–0.0384 to 0.1650) | 0.211 |  | 0.1635 (0.0454 to 0.2816) | 0.009 |
| For females | –0.1349 (–0.2037 to –0.0661) | <0.001 |  | –0.0404 (–0.0577 to –0.0230) | <0.001 |  | –0.0530 (–0.1241 to 0.0182) | 0.138 |
| Second difference (Reference: males) | –0.2351 (–0.3719 to –0.0982) | 0.002 |  | –0.1037 (–0.2086 to 0.0012) | 0.053 |  | –0.2165 (–0.3626 to –0.0703) | 0.005 |
| *Food support* |  |  |  |  |  |  |  |  |
| For males | –0.0808 (–0.1277 to –0.0338) | 0.002 |  | 0.0047 (–0.0622 to 0.0715) | 0.887 |  | –0.0442 (–0.0787 to –0.0097) | 0.014 |
| For females | –0.0152 (–0.0632 to 0.0329) | 0.522 |  | 0.0063 (–0.0216 to 0.0341) | 0.647 |  | 0.0019 (–0.0335 to 0.0373) | 0.915 |
| Second difference (Reference: males) | 0.0656 (0.0038 to 0.1274) | 0.038 |  | 0.0016 (–0.0685 to 0.0717) | 0.963 |  | 0.0460 (–0.0043 to 0.0964) | 0.071 |

1. **Zambia**

|  | Condomless sex |  |  | Multiple sexual partnerships | |  | High-risk sex |  |
| --- | --- | --- | --- | --- | --- | --- | --- | --- |
|  | AME (95% CI) | p value |  | AME (95% CI) | p value |  | AME (95% CI) | p value |
| *Social transfer* |  |  |  |  |  |  |  |  |
| For males | 0.0100 (–0.0783 to 0.0983) | 0.817 |  | 0.0175 (–0.0579 to 0.0929) | 0.637 |  | 0.0168 (–0.0442 to 0.0779) | 0.575 |
| For females | –0.0854 (–0.1610 to –0.0098) | 0.028 |  | 0.0163 (–0.0127 to 0.0452) | 0.258 |  | –0.0669 (–0.1264 to –0.0075) | 0.029 |
| Second difference (Reference: males) | –0.0954 (–0.1994 to 0.0086) | 0.071 |  | –0.0012 (–0.0817 to 0.0793) | 0.975 |  | –0.0838 (–0.1598 to –0.0077) | 0.032 |
| *Educational support* | |  |  |  |  |  |  |  |
| For males | –0.0772 (–0.1831 to 0.0288) | 0.146 |  | 0.0164 (–0.0661 to 0.0989) | 0.686 |  | –0.0105 (–0.0926 to 0.0716) | 0.795 |
| For females | –0.1104 (–0.1990 to –0.0217) | 0.017 |  | –0.0280 (–0.0399 to –0.0160) | <0.001 |  | –0.0583 (–0.1292 to 0.0125) | 0.102 |
| Second difference (Reference: males) | –0.0332 (–0.1737 to 0.1073) | 0.631 |  | –0.0444 (–0.1280 to 0.0393) | 0.285 |  | –0.0479 (–0.1564 to 0.0607) | 0.373 |
| *Food support* |  |  |  |  |  |  |  |  |
| For males | –0.0554 (–0.1851 to 0.0743) | 0.388 |  | 0.0463 (–0.1061 to 0.1987) | 0.537 |  | –0.0444 (–0.1112 to 0.0223) | 0.183 |
| For females | –0.0118 (–0.1497 to 0.1260) | 0.861 |  | 0.0093 (–0.0582 to 0.0768) | 0.779 |  | –0.0036 (–0.1104 to 0.1032) | 0.945 |
| Second difference (Reference: males) | 0.0436 (–0.1333 to 0.2204) | 0.616 |  | –0.0370 (–0.2048 to 0.1307) | 0.653 |  | 0.0408 (–0.0913 to 0.1730) | 0.530 |

1. **Zimbabwe**

|  | Condomless sex |  |  | Multiple sexual partnerships | |  | High-risk sex |  |
| --- | --- | --- | --- | --- | --- | --- | --- | --- |
|  | AME (95% CI) | p value |  | AME (95% CI) | p value |  | AME (95% CI) | p value |
| *Social transfer* |  |  |  |  |  |  |  |  |
| For males | –0.0355 (–0.0704 to –0.0006) | 0.047 |  | –0.0028 (–0.0396 to 0.0339) | 0.875 |  | –0.0078 (–0.0345 to 0.0189) | 0.552 |
| For females | 0.0226 (–0.0228 to 0.0680) | 0.315 |  | 0.0177 (–0.0120 to 0.0473) | 0.232 |  | 0.0273 (–0.0136 to 0.0682) | 0.182 |
| Second difference (Reference: males) | 0.0581 (0.0026 to 0.1137) | 0.041 |  | 0.0205 (–0.0299 to 0.0709) | 0.410 |  | 0.0351 (–0.0143 to 0.0845) | 0.156 |
| *Educational support* | |  |  |  |  |  |  |  |
| For males | –0.0118 (–0.0810 to 0.0575) | 0.729 |  | –0.0031 (–0.0673 to 0.0611) | 0.922 |  | –0.0111 (–0.0612 to 0.0390) | 0.652 |
| For females | –0.1624 (–0.2309 to –0.0938) | <0.001 |  | –0.0093 (–0.0343 to 0.0157) | 0.450 |  | –0.1098 (–0.1591 to –0.0604) | <0.001 |
| Second difference (Reference: males) | –0.1506 (–0.2442 to –0.0570) | 0.003 |  | –0.0062 (–0.0785 to 0.0661) | 0.861 |  | –0.0987 (–0.1674 to –0.0299) | 0.007 |
| *Food support* |  |  |  |  |  |  |  |  |
| For males | –0.0215 (–0.0651 to 0.0222) | 0.321 |  | 0.0228 (–0.0261 to 0.0717) | 0.346 |  | 0.0028 (–0.0347 to 0.0403) | 0.879 |
| For females | –0.0440 (–0.1008 to 0.0129) | 0.124 |  | –0.0068 (–0.0336 to 0.0201) | 0.607 |  | 0.0028 (–0.0502 to 0.0558) | 0.914 |
| Second difference (Reference: males) | –0.0225 (–0.0899 to 0.0449) | 0.498 |  | –0.0296 (–0.0848 to 0.0256) | 0.280 |  | –0.0000 (–0.0646 to 0.0645) | 1.000 |
